# Gardiquimod Nanoemulsion Targets Cutaneous Leishmaniasis Lesions Reducing Systemic Toxicity and Parasite Burden

**DOI:** 10.1002/adhm.71415

**Published:** 2026-07-06

**Authors:** Carmen Palomino‐Cano, M. Carmen Mera‐Delgado, Esther Moreno, Esther Larrea, Lecnia Aguirre, Jonathan Miguel Zanatta, Sónia Barros‐Carvalho, Iola F. Duarte, Ricardo Silvestre, Juan M. Irache, Javier Carrión, Socorro Espuelas

**Affiliations:** ^1^ Department of Pharmaceutical Sciences School of Pharmacy and Nutrition University of Navarra Pamplona Spain; ^2^ Department of Molecular Biology. School of Sciences University of Navarra Pamplona Spain; ^3^ Institute For Health Research (IdiSNA) Pamplona Spain; ^4^ Institute of Biomedical Sciences University of São Paulo São Paulo Brazil; ^5^ Life and Health Sciences Research Institute (ICVS) School of Medicine University of Minho Braga Portugal; ^6^ ICVS/3B's‐PT Government Associate Laboratory Braga/Guimarães Portugal; ^7^ LAQV‐REQUIMTE Department of Chemistry University of Aveiro Aveiro Portugal; ^8^ Department of Animal Health, Faculty of Veterinary Sciences, Complutense University of Madrid Madrid Spain

**Keywords:** cutaneous leishmaniasis, drug delivery, immunomodulation, macrophages, nanoemulsion, TLR agonist

## Abstract

Cutaneous leishmaniasis (CL) remains difficult to treat because *Leishmania* parasites persist within dermal macrophages and suppress their microbicidal activity, promoting chronic infection. Toll‐like receptor (TLR) agonists can restore macrophage effector functions, but their therapeutic use is limited by systemic toxicity. Here, we developed a lipid‐based nanoemulsion (NE) for the systemic delivery of the TLR7 agonist gardiquimod (GDQM) to improve macrophage targeting and reduce off‐target inflammatory effects. Following intravenous administration, GDQM‐NEs efficiently accumulated in dermal lesions and significantly reduced systemic cytokine release compared with free GDQM, resulting in an improved safety profile. Importantly, nanoencapsulation preserved GDQM antileishmanial activity achieving an approximately 2‐log reduction in parasite burden. Treatment induced both Th1‐associated cytokines (IFN‐γ and IL‐12p70) and regulatory responses, including IL‐10 production and FoxP3^+^ regulatory T‐cell expansion. Despite efficient lesion accumulation, CD86 upregulation was detected in macrophages from draining lymph nodes (dLNs) but not at the lesion site, indicating limited local macrophage activation. These findings indicate that GDQM‐NEs effectively overcome major delivery and safety barriers associated with systemic TLR7 agonist administration, whereas the lesion microenvironment and TLR‐driven regulatory mechanisms remain key constraints on therapeutic efficacy. Combining TLR agonists with strategies that counteract these local suppressive pathways may further enhance treatment outcomes in CL.

## Graphical abstract

Nanoemulsion delivery of a TLR7 agonist enhances safety and promotes colocalization with *Leishmania*‐infected macrophages in skin lesions, enabling a ∼2‐log reduction in parasite burden. However, tissue‐level constraints and TLR‐driven regulatory feedback generate a mixed immune response that limits complete parasite clearance.

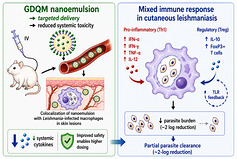

## 1 Introduction

Leishmaniasis is classified as a neglected tropical disease and is transmitted through the bite of infected female phlebotomine sandflies, predominantly impacting socioeconomically vulnerable populations in tropical and subtropical areas. The disease presents with a wide range of clinical outcomes, from localized cutaneous ulcers that may resolve spontaneously to severe visceral forms that can be lethal, representing a substantial public health challenge in endemic regions [1]. The causative agents of leishmaniasis are flagellated protists of the *Leishmania* genus that are obligate intracellular parasites that survive and replicate within macrophages. It is a paradoxical evolutionary decision given that these cells are specialized in pathogen destruction. Macrophages exhibit remarkable phenotypic and functional plasticity and are traditionally classified into two extreme activation states: M1, which are pro‐inflammatory, microbicidal, and produce nitric oxide (NO) and reactive oxygen species (ROS); and M2, which are anti‐inflammatory, involved in tissue repair, and generally permissive to intracellular pathogens. *Leishmania* exploits this plasticity by skewing macrophages toward an M2‐like phenotype, subverting innate microbicidal mechanisms, and creating a cellular environment that supports long‐term parasite survival [2].

Given the central role of macrophages in the outcome of infection, strategies aimed at reprogramming their activation state have emerged as promising alternatives to conventional chemotherapy, with the added benefit of circumventing pharmacological resistance [3, 4]. (TLRs), which detect microbial ligands and trigger signaling cascades that promote classical macrophage activation, pro‐inflammatory cytokine production, and enhanced antimicrobial functions, are particularly relevant [5]. Although TLRs have been described as having a dual role in *Leishmania* infection [6], numerous studies across parasite species and TLR agonists show that their selective activation can effectively reprogram macrophages, counteract the parasite‐driven M2 bias, and improve parasite control [7, 8, 9].

Within this context, TLR7, a receptor for single‐stranded RNA expressed in macrophages, monocytes, dendritic cells, plasmacytoid dendritic cells (pDCs), and B lymphocytes, has attracted particular interest due to its ability to induce coordinated pro‐inflammatory and type I interferon responses. Compared to other TLR pathways, TLR7 activation provides a balanced induction of antimicrobial cytokines while avoiding the tolerogenic profiles associated with TLR2 [10], the parasite‐supporting effects reported for TLR3 [11], or the excessive systemic inflammation linked to TLR4 [12], making it a particularly suitable target for immunomodulation in this context. Indeed, one of its agonists, imiquimod (IMQ), has been extensively used at both preclinical and clinical levels in CL [7, 13, 14, 15, 16, 17, 18], and more potent derivatives, such as GDQM, have been developed. GDQM strongly activates NF‐κB and IRF7 signaling, promoting type I interferon production and robust M1‐skewing responses. Although GDQM has demonstrated immunotherapeutic potential in cancer [19, 20] and as a prophylactic vaccine adjuvant against *L. donovani* [21, 22], it has not yet been evaluated as a stand‐alone therapy in established infections or for its ability to reverse parasite‐induced macrophage immunosuppression.

A major challenge limiting the therapeutic use of potent TLR7 agonists is their systemic immunotoxicity. Encapsulation offers an effective strategy to overcome this limitation because i) nanocarriers markedly reduce off‐target systemic activation while promoting passive accumulation within macrophages, which constitute the natural niche of *Leishmania* and the final destination of intravenously administered particles; and ii) they enable controlled and sustained drug release, ensuring effective intracellular delivery without provoking widespread inflammation.

However, in CL, infected macrophages reside deep in the dermis, which severely limits drug accumulation and compromises the efficacy of local treatments. Nanoparticles, far from solving this problem, cannot reach these compartments: even under the inflammatory and tissue‐disrupted conditions of CL, no nanoparticle, not even the smallest, penetrates deeply enough to access the dermal regions where *Leishmania*‐infected macrophages reside [23]. As a result, selective drug delivery to these cells remains a major bottleneck, and therapeutic efficacy critically depends on achieving adequate concentrations at the intracellular target site, making the route of administration a central determinant of success [24]. For this reason, intralesional injection is the preferred option for CL and is recommended by the World Health Organization, as it delivers high drug levels directly into deep skin layers. Nevertheless, this route is painful, operator‐dependent, and unsuitable for large, multiple, or anatomically complex lesions. In such scenarios, including diffuse forms of CL, systemic therapy becomes essential [25]. Thus, the development of a systemically deliverable nanocarrier capable of safely transporting and enriching TLR7 agonists within infected macrophages addresses a key unmet therapeutic need. Importantly, whether systemically administered nanocarriers can effectively access parasite‐infected macrophages within established lesions, and critically, whether they reach the intracellular niche where the parasite resides, remains largely unresolved. Direct evidence of nanoparticle co‐localization with infected cells in vivo is still lacking, representing a major gap in our understanding of drug delivery in CL.

Based on this rationale, we load GDQM in a previous laboratory‐optimized lipid NE [26] composed of medium‐chain triglycerides, oleic acid, and polyoxyethylene glycol (PEG)‐40 stearate, designed for intravenous delivery. We first conducted an in‐depth in vitro analysis with both blank and GDQM‐loaded NEs, assessing their effects on macrophage activation, immunometabolic reprogramming, and infection control. Subsequently, we conducted biodistribution studies in a murine ear model of CL to assess the spatial colocalization of intravenously delivered NEs with parasite‐infected macrophages. Finally, we designed in vivo therapeutic experiments to evaluate the potential of GDQM‐loaded NEs as a targeted immunotherapeutic approach against cutaneous leishmaniasis.

## 2 Material and Methods

### 2.1 Materials

Gardiquimod was purchased from MedChemExpress (Monmouth Junction, NJ, USA). 1,1′‐dioctadecyl‐3,3,3′,3′‐tetramethylindodicarbocyanine 4‐chlorobenzenesulfonate salt (DiD) was obtained from Interchim (Montluçon, France). Myrj S40 (PEG‐40 stearate) was kindly provided by Croda (Snaith, United Kingdom). Lipoid S100 (soybean lecithin containing >94% phosphatidylcholine, PC) was kindly supplied by Lipoid GmbH (Ludwigshafen, Germany). Suppocire CM (a medium–long‐chain triglyceride mixture, C10–C18) was kindly provided by Gattefosé (Saint‐Priest, France). Oleic acid (OA) was purchased from TCI (Zwijndrecht, Belgium). 3‐(4,5‐dimethylthiazol‐2‐yl)‐2,5‐diphenyltetrazolium bromide (MTT) was obtained from Sigma (St. Louis, MO, USA). Phosphate‐buffered saline (PBS), Dulbecco's Modified Eagle Medium (DMEM), Roswell Park Memorial Institute Medium (RPMI) 1640, fetal bovine serum (FBS), and penicillin–streptomycin were purchased from Gibco (Gaithersburg, MD, USA). IFN‐γ and IL‐4 from mouse were purchased from Immunotools (Friesoythe, Germany). LPS from *E.Coli* (0111:B4) was obtained from InvivoGen (San Diego, USA). Ultrapure water (>18 MΩ·cm resistivity) was produced using an Ultramatic Type I system (Wasserlab, Spain). Antibodies used in this study were supplied by BioLegend (San Diego, CA, USA). All other reagents and chemicals were of analytical grade.

### 2.2 Preparation of Blank, GDQM‐Loaded, and Fluorescent NEs

The composition of this NE was previously optimized in our laboratory for IMQ [26], and in the present study, the same procedure was applied for the encapsulation of GDQM. Briefly, GDQM (6 mg) was dissolved in OA (30 µL; solubility: 220 mg/mL, experimentally determined) and subsequently mixed with Suppocire CM (190 mg) upon heating at 45°C to obtain a homogeneous lipid phase. Soybean lecithin (115 mg), previously dissolved in dichloromethane, was then incorporated into the lipid phase. Blank nanoemulsions were prepared following the same procedure without GDQM. Next, 10 mL of the aqueous phase, composed of Myrj S40 (10 mm in PBS), was added to the system, and the resulting mixture was sonicated for 10 min using an Ultrasonic Processor BH‐300N operating at 50% amplitude (equivalent to 75 W output power). Following sonication, the NE formulations were kept on ice for 1 h to allow proper formation and stabilization of the lipid carriers. To eliminate unencapsulated GDQM, the dispersions underwent a short centrifugation at 5000 *× g* for 10 min at 4°C. The supernatant was then transferred to Amicon centrifugal filter units (30 kDa MWCO, Vivaspin, Sartorius) and centrifuged at 5000 *× g* for 30 min at 4°C to concentrate and further purify the NEs. The resulting purified formulations were stored as aqueous dispersions at 4°C until further experimental use.

For the preparation of fluorescent NEs, the lipophilic fluorophore DiD was incorporated into the melted lipid phase prior to the addition of the aqueous phase. DiD was dissolved in dichloromethane and added at 1% w/w relative to the total lipid content (3 mg per formulation).

### 2.3 NEs Characterization

#### 2.3.1 Size, PDI and Zeta Potential

Particle size and polydispersity index (PDI) were determined by dynamic light scattering (DLS), while zeta potential was measured using electrophoretic light scattering (ELS). For these measurements, samples were diluted in ultrapure water. Analyses were performed using a Malvern Zetasizer Pro. Results are presented as the mean ± standard deviation of three independent measurements at 25°C.

#### 2.3.2 Encapsulation Efficiency of GDQM and DiD

The content of GDQM and DiD was quantified by UV–visible spectroscopy (Agilent Technologies 8453) using standard curves prepared in HCl:ACN (1:9). Concentration ranges were 2.5–25 µg/mL for GDQM and 1–15 µg/mL for DiD. NEs were disrupted with HCl:acetonitrile and centrifuged at 20,000 ×*g* for 15 min (Centrifuge 3K30, Sigma). The supernatant was collected, and absorbance was measured at 322 nm for GDQM and 650 nm for DiD.

#### 2.3.3 Stability and Drug Release Studies

Formulation stability was assessed at 4°C and 37°C by monitoring particle size and PDI over time using DLS, representing storage and physiological conditions, respectively, in line with commonly accepted practices in pharmaceutical stability studies (ICH Q1A(R2)). For stability studies in biological media, DMEM supplemented with 10% (v/v) heat‐inactivated FBS and 1% penicillin/streptomycin (complete DMEM, DMEMc) was selected due to its plasma‐like composition, consistent with current recommendations to evaluate nanomaterial behavior in relevant biological environments (ISO/TR 13014). To evaluate potential aggregation, formulations were diluted at a 1:10 ratio in DMEMc. Turbidity was monitored over 2 h (as an indicator of aggregation) by measuring absorbance at 605 nm with an Agilent 8453 spectrophotometer.

In vitro release of GDQM from NEs was evaluated using a dialysis‐based method under sink conditions at 37°C. The formulation was placed in 3.5 kDa Flot‐A‐Lyzer dialysis devices and immersed in 5% (v/v) Tween 80 solution. At specific time points (6, 24, and 36 h), 100 µL samples were collected from the receptor medium and replaced with fresh solution. Drug concentration was measured by UV–vis spectroscopy at 325 nm (Agilent 8453), using a calibration curve in 5% Tween 80. The cumulative drug release was calculated based on the concentration in the receptor, total volume, and initial GDQM content.

#### 2.3.4 SEM Images

The morphology and shape of the NEs were analyzed using scanning electron microscopy (SEM) at 3 kV and a 4 mm working distance. For this, 4 µL of the fresh formulations were placed on flat stubs 12.7 mm in diameter. After allowing the droplets to dry at room temperature, the samples were coated with a gold layer using the Emitech K550 gold coater (Quorum Technologies). Finally, the samples were examined using a FESEM Zeiss Sigma 300 VP microscope (Zeiss Microscopy).

### 2.4 Animals and Parasites

Female BALB/c mice (Envigo), with an average weight of 20 g, were used and maintained under standard animal facility conditions. The animals were housed in groups of six per cage, in temperature‐controlled rooms (22°C) with 12‐h light/dark cycles and had free access to food and water. All experimental procedures were approved by the Animal Experimentation Ethics Committee of the University of Navarra (protocol number 123‐21) and carried out in accordance with the current European regulations on the use of animals in research (Directive 2010/63/EU).

For the experiments, a clonal line of *Leishmania major* (Friedlin VI) genetically modified to express the fluorescent protein mCherry, integrated into the 18S ribosomal gene locus, was used. This strain was kindly provided by Dr. José M. Requena and Dr. José Carlos Solana (Severo Ochoa Molecular Biology Center, Madrid, Spain). Parasites were cultured at 26°C in Schneider's Drosophila medium (Sigma), maintained under continuous agitation, and supplemented with 20% (v/v) heat‐inactivated FBS, 1% penicillin/streptomycin (Sigma), and 50 µg/mL blasticidin as a selection agent for mCherry expression. Cultures were grown in 25 cm^2^ flasks. Parasite virulence was maintained by periodic passages in BALB/c mice and reisolation from infected tissues.

### 2.5 Isolation and Differentiation of Macrophages from Murine Bone Marrow (BMDM)

Femurs and tibias from the hind limbs were aseptically collected and briefly rinsed in 70% ethanol. The epiphyses were removed, and bone marrow was flushed from the medullary cavity using a 25 G needle and DMEMc. The bone marrow from each leg was transferred into a 10 cm diameter Petri dish containing 20 mL of DMEMc supplemented with 20% (v/v) filtered L929 cell‐conditioned medium, serving as a source of macrophage colony‐stimulating factor (M‐CSF). Cultures were incubated at 37°C with 5% CO_2_ for 7 days. To maintain M‐CSF levels, 10 mL of fresh L929‐conditioned medium was added every 3 days. On day 7, adherent macrophages were washed twice with PBS, detached using TPP cell scrapers, and collected for downstream experiments.

### 2.6 Isolation of Metacyclic Promastigotes and Infection of Macrophages and Mice

For both in vitro and in vivo infections, metacyclic promastigotes were isolated using the classical peanut agglutinin (PNA) method described by Sacks et al. [27]. Briefly, 10 mL of stationary‐phase promastigote cultures were centrifuged at 3000 *× g* for 10 min and washed twice with RPMI medium. Parasites were then incubated for 30 min with PNA (500 µg per 5×10^8^ parasites) and centrifuged at 50 *× g* for 5 min. Non‐agglutinated promastigotes, enriched in the metacyclic form, were collected from the supernatant and used for subsequent infections.

For in vivo infection, metacyclic promastigotes were washed twice with sterile PBS and injected intradermally into the mouse ear (10^4^ parasites in 10 µL). For in vitro assays, 7‐day differentiated BMDM were infected at a multiplicity of infection of 10 parasites per cell. The infection was allowed to proceed for 24 h to enable parasite internalization and differentiation into amastigotes, as previously described [28]. After this period, four PBS washes were performed to remove extracellular promastigotes, and fresh medium containing various concentrations of NEs was added for downstream experiments.

### 2.7 In Vitro Assays in Non‐Infected and *L. major‐*Infected Macrophages

#### 2.7.1 Cytotoxicity of NE

To assess the cytotoxicity of NE, BMDM were seeded at a density of 9 × 10^4^ cells per well in 96‐well plates (200 µL) and treated with increasing concentrations of the formulation and maintained at 37°C with 5% CO_2_ for 48 h. Cell viability was evaluated using the MTT assay, which measures mitochondrial metabolic activity as an indicator of live, functional cells. Following treatment, MTT reagent was added to each well at a final concentration of 0.5 mg/mL and incubated for 4 h to allow formazan crystal formation. After incubation, supernatants were carefully removed, and 100 µL of DMSO was added to solubilize the formazan. Absorbance was measured at 540 nm using a microplate reader (Agilent Technologies 8453). Untreated cells served as viability controls and were used to normalize the data, which are expressed as a percentage of viable cells relative to the control. Results represent the mean ± standard deviation (SD) of at least two independent experiments, each conducted in triplicate.

#### 2.7.2 Cellular Uptake of DiD‐Labeled NEs

To evaluate the internalization of NEs by BMDM, macrophages were seeded at a density of 1 × 10^6^ cells per well in 24‐well plates (1 mL) and incubated for 3 h at 37°C with DiD‐labeled NEs at concentrations of 0.02, 0.2, 2, and 20 µg/mL DiD. Following incubation, cells were washed with PBS and incubated with 2.5 mM EDTA in PBS for 10 min at 37°C to facilitate detachment. Cells were then gently collected using a sterile cell scraper. Flow cytometry was performed using a CytoFlex LX flow cytometer (Beckman Coulter, Indianapolis, USA), and data were analyzed with FlowJo V10 software. NE uptake was quantified as the percentage of DiD‐positive cells and the relative mean fluorescence intensity (rMFI), which was calculated as the ratio of the mean fluorescence intensity of the sample to that of the control. Results are presented as the mean ± SD of three independent experiments.

In parallel, qualitative assessment of uptake was carried out using fluorescence microscopy. BMDMs were seeded on LabTek II chamber slides at a density of 1 × 10^5^ cells per well and treated under the same conditions. After incubation with DiD‐labeled NEs, cells were stained with LysoTracker Green DND‐26 (Lumiprobe) at a final concentration of 50 nM in PBS for 30 min at 37°C to label acidic compartments. Nuclear staining was performed using 1 µm DAPI for 20 min. Cells were washed with PBS and imaged live using fluorescence microscopy (Confocal Zeiss LSM 880).

#### 2.7.3 Phenotypic Characterization of NE‐Treated Macrophages

The immune profiling of BMDMs was carried out using both RT‐qPCR and flow cytometry. IFN‐γ + LPS (100 UI/mL and 100 ng/mL, respectively) and IL‐4 (20 ng/mL) were used as positive controls for M1 and M2 macrophages. The same concentrations of IFN‐γ and IL‐4 were applied when NEs were combined with co‐stimulatory cytokines.

For gene expression analysis of macrophage polarization markers, BMDMs were seeded at 3 × 10^5^ cells per well in 48‐well plates (0.5 mL). After 6 h of treatment with NEs, total RNA was extracted using the Maxwell RSC simplyRNA kit (Promega) following the manufacturer's protocol. RNA quantity and purity were assessed with a NanoPhotometer NP80 (Implen). Subsequently, RNA was reverse transcribed into complementary DNA (cDNA) using SuperScript III Reverse Transcriptase (Invitrogen). Quantitative real‐time PCR (qRT‐PCR) was performed with iQ SYBR Green Supermix (Bio‐Rad) on a CFX96 Real‐Time PCR Detection System (Bio‐Rad). Specific primers targeting genes related to macrophage polarization are detailed in Table S1. Gene expression levels were normalized to ß‐actin and calculated using the ΔΔCt method [29].

By flow cytometry, macrophage activation was evaluated through the expression of surface markers. Following the uptake protocol, 1 million BMDMs were seeded per well in 24‐well (1 mL) plates and treated for 48 h. Cells were then incubated with 2.5 mm EDTA for 30 min at 37 °C and gently detached using a cell scraper. The collected cells were transferred to V‐bottom 96‐well plates for staining. Cells were stained with fluorochrome‐conjugated antibodies against F4/80‐PercpC5.5 (clone BM8, Invitrogen), CD206‐APC (clone C068C2, Biolegend), and CD86‐PE (clone GL‐1, Biolegend). Staining was performed on ice for 15 min, followed by washing with FACS buffer (PBS with 2% FBS). Cells were then fixed with 2% paraformaldehyde for 10 min, resuspended in FACS buffer, and analyzed on a BD LSR II flow cytometer. Data were processed and analyzed using FlowJo V10 software. Results are expressed as rMFI, calculated for each sample as the mean fluorescence intensity of the total population divided by that of the corresponding control. Values represent the mean ± SD of at least two independent experiments, each conducted in quadruplicate.

#### 2.7.4 Functional Responses of NE‐Treated Macrophages: ROS, NO, and Cytokine Production

In the experiments measuring ROS, NO, and cytokine production, the same M1 (IFN‐γ + LPS) and M2 (IL4) positive controls previously described for phenotypic assays were also included. (ROS) production in macrophages exposed to NEs was quantified by flow cytometry using mitochondrial‐specific fluorescent probes. 1 million BMDMs were seeded per well in 24‐well (1 mL). After 48 h of treatment, cells were detached using 2.5 mM EDTA and gently collected with a cell scraper. The cell suspension was transferred to 96‐well V‐bottom plates, washed with PBS, and incubated with 60 µL of staining solution containing MitoTracker Deep Red (Invitrogen, M22426) to label viable mitochondria and MitoSOX Red (Invitrogen, M3008) to detect mitochondrial superoxide. The staining was performed for 30 min at 37°C, after which cells were immediately analyzed on a BD LSR II flow cytometer. Data were processed and analyzed using FlowJo V10 software. Results represent the mean ± SD of at least two independent experiments, each conducted in quadruplicate.

The secretion of (NO) and cytokines was measured in the supernatants collected from the same macrophage cultures after 48 h of NEs exposure. Nitrite levels, as an indicator of NO production, were assessed via the Griess assay. In brief, equal volumes (50 µL) of culture supernatant and Griess‐Ilosvay reagents A and B (Panreac) were combined in microplate wells and incubated for 30 min at room temperature. Absorbance was measured at 540 nm (Agilent Technologies 8453), and nitrite concentrations were calculated using a sodium nitrite (NaNO_2_) standard curve. Concentrations of IL‐12p70, IL‐10, and TNF‐α were determined using commercially available ELISA kits (BD Biosciences) according to the manufacturer's instructions.

#### 2.7.5 Metabolic Profiling

To evaluate the bioenergetic status of macrophages exposed to GDQM‐loaded NEs, the Seahorse XF Cell Mito Stress Test (Agilent Seahorse Bioscience) was performed. Briefly, 5 × 10^4^ BMDMs were seeded per well and treated with GDQM NEs at a concentration of 10 µm for 48 h. Immediately before the assay, cells were washed and incubated for 1 h in Seahorse XF assay medium (DMEM base) adjusted to pH 7.4 and supplemented with 10 mm glucose, 1 mm sodium pyruvate, and 2 mm glutamine. Oxygen comsumption rate (OCR) and extracellular acidification rate (ECAR) were measured using an XF96 Seahorse Extracellular Flux Analyzer following the manufacturer's instructions. The following metabolic modulators were injected at their final working concentrations: oligomycin (1 µm), carbonylcyanide‐p‐trifluoromethoxiphenylhydrazone (FCCP, 1 µm), rotenone/antimycin A (1 µm each), and 2‐deoxyglucose (2‐DG, 50 mm). Data were normalized to total protein content per well. Results are expressed as mean ± SD from at least two independent experiments performed in duplicate.

To further characterize the metabolic changes induced by NEs treatment beyond the Seahorse bioenergetic analysis, metabolomic profiling of cell culture supernatants was performed using nuclear magnetic resonance (NMR) spectroscopy. Supernatants (300 µL), collected from cultures at a density of 1 × 10^6^ BMDM/mL, were transferred to methanol‐prewashed microtubes, and 900 µL of cold 100% methanol was added. After incubating for 20 min at −20°C to precipitate proteins, the samples were centrifuged at 14 000 *× g* for 15 min at 4°C, and the clarified supernatants were vacuum‐dried. Prior to NMR analysis, dried extracts were reconstituted in 550 µL of deuterated phosphate buffer containing 0.2 mm trimethylsilylpropanoic acid (TSP‐*d_4_*) as an internal standard, and transferred to 5 mm NMR tubes. NMR spectra were acquired on a Bruker Avance III HD 500 NMR spectrometer (University of Aveiro, Portuguese NMR Network) operating at 500.18 MHz for ^1^H observation, at 298 K. One‐dimensional (1D) ^1^H spectra were recorded with 48 k points, 6009.6 Hz spectral width, a 5 s recycle time, and 256 scans, using the “noesypr1d” pulse program. Spectral processing was carried out in TopSpin 4.0.3 (Bruker BioSpin, Rheinstetten, Germany), and consisted of exponential multiplication (LB 0.3 Hz), zero‐filling to 64 k data points, manual phasing, baseline correction, and calibration to the TSP‐*d_4_* signal (0 ppm). Two‐dimensional (2D) NMR spectra, namely ^1^H‐^1^H TOCSY and *J*‐resolved, were recorded for selected samples to aid metabolite identification. Signal assignment was based on matching 1D and 2D spectral information to reference spectra in Chenomx 9.0 (Edmonton, AB, Canada) and BBIOREFCODE‐2–0–0 (Bruker Biospin). For quantitative measurement of metabolic variations, selected signals representative of individual metabolites were integrated using Amix‐Viewer 3.9.15 (Bruker Biospin). Signal areas were then used to calculate the variations in cell‐conditioned media relative to acellular media, incubated under the same conditions.

#### 2.7.6 Quantification of Parasite Burden

Parasite burden was quantified by RT‐qPCR. Similar to the gene expression studies, 3 × 10^5^ BMDMs were seeded per well in 48‐well plates (0.5 mL) and infected with *L. major* (section 2.6). After 24 hours of infection, the macrophages were treated with increasing concentrations of GDQM‐loaded NEs (0.1, 1, and 10 µm) or the same amounts of blank NEs, with or without co‐stimuli (IFN‐γ, 100 UI/mL) and IL‐4 (20 ng/mL). Forty‐eight hours later, total RNA was extracted following the same protocol used for gene expression analysis. Relative quantification of infection was performed using the *Leishmania* 18S ribosomal RNA gene as the target and β‐actin as the housekeeping gene for the host cells. The sequences of the primers used for the amplification of 18S rRNA are in Table S1.

### 2.8 Biodistribution of DiD‐Labeled NE in *L. major*‐Infected Mice

#### 2.8.1 In Vivo and Ex Vivo Fluorescence Imaging

To evaluate the biodistribution of NEs in a model of cutaneous leishmaniasis, BALB/c mice were infected in the ear as previously mentioned (Section 2.6) with mCherry‐expressing *L. major* promastigotes and allowed to develop lesions for 5 weeks. Fluorescently labeled NEs (DiD‐NE) were administered intravenously (1 mg/kg DiD, 100 µL) via the tail vein. Mice were euthanized at 24‐, 72‐, and 96‐h post‐administration by cervical dislocation. Whole‐body in vivo fluorescence was imaged using the IVIS Spectrum imaging system (PerkinElmer) under isoflurane anesthesia. Subsequently, major organs (liver, spleen, kidneys, dLNs, and ears) were harvested and imaged ex vivo under the same acquisition settings to quantify DiD fluorescence. Fluorescence intensity was expressed as radiant efficiency and analyzed using Living Image software.

#### 2.8.2 Co‐Localization of DiD‐NE and Parasites, and Immune Cell Phenotyping in Infected Ears

To investigate NE accumulation at the site of infection and potential co‐localization with *L. major‐*mCherry parasites, infected ears were processed into single‐cell suspensions. Briefly, ears were dissected and minced with fine scissors, then incubated in 2 mL of digestion buffer containing collagenase (1 mg/mL) and DNase I (0.1 mg/mL) at a 10:1 ratio for 1 h at 37°C with gentle agitation. Following enzymatic digestion, tissue was mechanically dissociated using a 70 µm cell strainer and the plunger of a 2 mL syringe. The resulting suspension was centrifuged, and cell pellets were resuspended in PBS. Two million cells per well were plated in V‐bottom 96‐well plates for immunostaining.

Before surface staining, cells were incubated for 20 min on ice with purified anti‐mouse CD16/32 antibody (clone 93 Biolegend) in 50 µL of FACS buffer (PBS with 2% FBS and 2.5 mM EDTA) to block Fc receptors and minimize nonspecific binding. Surface markers included: Ly6G‐BV510 (clone 1A8) for neutrophils, CD11b‐PE‐Cy7 (clone M1/70) for macrophages, CD11c‐BV605 (clone N418) for dendritic cells, CD45‐FITC (clone QA17A26) for leukocytes, and CD86‐PE (clone BU63) as an activation marker. Cell viability was assessed using ZombieNIR‐APC‐Cy7. NE uptake was detected through DiD fluorescence, and parasite presence was identified by the mCherry signal. Samples were acquired using a CytoFlex LX flow cytometer (Beckman Coulter), and data were analyzed with FlowJo v10 software.

#### 2.8.3 Phenotypic Analysis of Immune Cells in dLNs

dLNs were collected from treated animals at the same time points to assess immune cell activation. Cell suspensions were obtained by mechanical dissociation, followed by filtration using a 70 µm cell strainer. Staining was done similarly to the ears. After Fc blocking with anti‐CD16/32, viability staining (ZombieNIR), and washing, cells were stained with the following antibody panel: CD11b‐PE‐Cy7 (clone M1/70), MHC class II–FITC (clone M5/114.15.2), B220–Pacific Blue (clone RA3‐6B2), CD11c‐BV605 (clone N418), and CD86‐PE (clone BU63). Using this panel, we identified B cells (B220^+^ CD11c^−^), pDCs (B220^+^ CD11c^+^), and myeloid antigen‐presenting cells (B220^−^ MHC II^+^ CD11b^+^). Samples were acquired using the CytoFlex LX flow cytometer, and data were analyzed using FlowJo v10.

### 2.9 Cytokine Quantification in Plasma

To evaluate the systemic early inflammatory response following intravenous treatment, plasma cytokine levels were quantified across all experimental conditions. Mice were assigned to seven groups, including untreated controls, free GDQM at two doses (0.5 and 1.5 mg/kg), GDQM‐loaded NEs at matched doses, and blank nanoemulsions administered at equivalent lipid concentrations.

Blood samples were collected from the saphenous vein (ear vein) into EDTA‐coated tubes at three time points: before treatment (baseline), and at 1 and 4 h post‐injection. Plasma was obtained by centrifugation at 2000 × *g* for 10 min at 4°C and stored at −80°C until analysis. Plasma levels of TNF‐α, IL‐6, and MCP‐1 were quantified using a Cytometric Bead Array (CBA) mouse inflammatory cytokine kit (Becton Dickinson), following the manufacturer's instructions. Samples were acquired on an Attune NxT acoustic focusing cytometer (Applied Biosystems, Waltham, MA, USA), and cytokine concentrations were calculated using standard curves generated for each analyte over a range of 20–5000 pg/mL. Type I interferon (IFN‐α) levels were measured separately using a commercial mouse IFN‐α ELISA kit (Thermo Fisher Scientific, BMS6027), according to the manufacturer's protocol.

### 2.10 Assessment of Therapeutic Efficacy in a Murine Model of CL

#### 2.10.1 Treatment Regimen and Infection Monitoring

To assess the in vivo therapeutic efficacy, *L. major*‐mCherry‐infected BALB/c mice were distributed into the same experimental conditions used for systemic analyses, including untreated controls, free GDQM at two doses (0.5 and 1.5 mg/kg), GDQM‐loaded nanoemulsions at matched doses, and blank nanoemulsions at equivalent lipid concentrations. All animals were bilaterally infected in both ears (*n* = 6–12). Starting two weeks after infection, treatments were administered intravenously once weekly. Infection progression was monitored weekly through two complementary approaches. First, parasite burden was quantified via in vivo fluorescence imaging using the IVIS Spectrum system (PerkinElmer), by measuring the mCherry signal from the parasites. Second, ear lesion size was monitored using a digital caliper to measure tissue swelling.

#### 2.10.2 Parasite Load Quantification by Limit Dilution Assay

At the end of the treatment period, ears and dLNs were collected to assess parasite burden. Quantification was performed using a limiting dilution assay, following the method described by Buffet et al., 1995 [30]. Tissue‐derived cell suspensions were serially diluted in Schneider's complete medium in 1:3 steps, in triplicate, in U‐bottom 96‐well culture plates, with a final volume of 150 µL per well. Plates were incubated at 26°C for seven days to allow viable amastigotes to transform into promastigotes. Wells were examined under a microscope for the presence of motile promastigotes. Parasite burden (P) was calculated as p = 3^n^ × dilution factor, where *n* is the number of the last well showing visible promastigotes. Results were expressed as the base‐10 logarithm of the geometric mean of the values obtained from the triplicate. For ears samples, values were normalized per milligram of tissue; for dLNs samples, they were normalized per one million cells.

#### 2.10.3 Preparation of Soluble Leishmania Antigen (SLA) for Antigen‐Specific Cell Restimulation Assays

SLA was prepared from stationary‐phase *L. major*‐mCherry promastigotes. A total of 50 mL of culture was centrifuged at 3000 *× g* for 10 min, washed twice with PBS, and resuspended in 0.5 mL PBS. The suspension was subjected to three freeze–thaw cycles using dry ice and a 37°C water bath. Next, the lysate was sonicated three times for 10 s at 80 % amplitude using an Ultrasonic Processor BH‐300N. The resulting material was centrifuged at 15 000 *× g* for 15 min at 4°C, and the protein content in the supernatant was quantified using the Pierce microBCA protein assay kit (Thermo Fisher Scientific).

#### 2.10.4 Restimulation and Cytokine/Flow Cytometric Analysis of dLNs Cells

Single‐cell suspensions were prepared from auricular dLNs of infected mice by mechanical disruption. Cells were cultured in complete RPMI 1640 (RPMIc) supplemented with 10% FBS, 1% antibiotic–antimycotic, 1% non‐essential amino acids (Gibco), 0.5% sodium pyruvate (Gibco), and 0.1% β‐mercaptoethanol (Gibco).

For *cytokine secretion* assays, 2 × 10^6^ cells per well were plated in U‐bottom 96‐well plates and restimulated for 48 h with SLA, (12 µg/mL). Supernatants were collected and IFN‐γ, IL‐12p70, IL‐10, TNF‐α (BD Biosciences), and IFN‐α (Invitrogen, detecting subtypes 2 and 4) were quantified by ELISA according to the manufacturers’ instructions.

For *flow cytometric analysis of T‐cell population*, parallel cultures were set up using the same cell density and stimulation conditions, but for 24 h, with brefeldin A (Merck) at 10 µg/mL) added only during the final 5 h to block cytokine secretion. After stimulation, cells were incubated with anti‐CD16/32 (clone 93) to block Fc receptors and stained for surface markers: CD3‐PE/Cy7 (clone 17A2), CD4‐FITC (clone GK1.5), CD25‐BV421 (clone PC61); and CD8‐APC‐H7 (clone 53–6.7). Intracellular staining for FoxP3‐Alexa Fluor 647 (clone QA20A67) was performed using the FoxP3/Transcription Factor Staining Buffer Set (BD Biosciences), according to the manufacturer's protocol. Samples were acquired on a CytoFLEX LX cytometer (Beckman Coulter) and analyzed with FlowJo v10.

#### 2.10.5 Gene Expression Analysis by RT‐qPCR in dLNs

To evaluate gene expression changes in lymphoid tissues, a portion of each dLN was collected immediately after dissection and preserved in homogenization buffer from the Maxwell RSC simplyRNA kit (Promega), followed by snap‐freezing to maintain RNA integrity. RNA extraction, reverse transcription, and quantitative PCR were performed following the same procedures described for in vitro experiments (2.7.3).

#### 2.10.6 Systemic Safety and Neuroinflammatory Assessment Following Repeated Treatment

To evaluate potential cumulative effects associated with repeated administration, systemic toxicity and neuroinflammatory responses were assessed at the end of the in vivo study. Body weight was recorded throughout the experimental period, with final measurements used as an indicator of overall health status.

At study endpoint, blood samples were collected from the submandibular vein and allowed to clot for 30 min. Serum was obtained by centrifugation at 3500 ×*g* for 10 min and used for biochemical analysis. Liver and kidney function markers, including alanine aminotransferase (ALT), aspartate aminotransferase (AST), alkaline phosphatase (ALP), gamma‐glutamyl transferase (GGT), urea, creatinine (CRE), albumin (ALB), total bilirubin (BILT), and C‐reactive protein (CRP), were quantified using a Cobas (Roche, Germany) automated biochemistry analyzer.

To assess potential neuroinflammatory effects, brains were collected, fixed by immersion in 4% paraformaldehyde for 72 h, and transferred to in 30% sucrose in PBS. Subsequently, brains were cut into 40‐µm‐thick coronal sections using a microtome and stored in cryoprotectant solution (2% dimethylsulphoxide, 20% glycerol in PBS) at −20°C until processing. For immunohistochemistry, free‐floating sections were washed in PBS, and endogenous peroxidase activity was quenched using 0.3% hydrogen peroxide in methanol. After blocking with PBS containing 0.5% Triton X‐100 and 5% normal goat serum, sections were incubated overnight at 4°C with a rabbit anti‐Iba1 antibody (1:1000; 019–19741, Wako). After washing, sections were incubated with biotinylated goat anti‐rabbit secondary antibody (1:500; E0432, Dako), followed by amplification using an HRP‐conjugated avidin–biotin complex (Vector Laboratories). Immunoreactivity was visualized using 3,3′‐diaminobenzidine (DAB Substrate Kit, Peroxidase (HRP), Vector Laboratories, SK‐4100), and sections were mounted, dehydrated through graded alcohols, cleared in xylene, and coverslipped with DPX mounting medium. Microglial activation was quantified using ImageJ.

### 2.11 Statistical Analysis

Statistical analyses were performed using GraphPad Prism (version 8.0.2). Data distribution was assessed using the Shapiro–Wilk normality test. For comparisons between two groups, unpaired two‐tailed Welch's t‐tests were used. For comparisons involving more than two groups, one‐way ANOVA followed by Tukey's multiple comparisons test was used for normally distributed data, whereas the Kruskal–Wallis test followed by Dunn's multiple comparisons test was applied for non‐normally distributed data. For grouped data involving two independent variables, two‐way ANOVA followed by Šídák's multiple comparisons test was used to compare selected groups within each condition. Statistical significance is indicated as follows: ^*^ *p* < 0.05, ^**^ *p* < 0.01, ^***^ *p* < 0.001, ^****^ *p* < 0.0001. The exact statistical tests used are indicated in the corresponding figure legends, including test statistics (t for t‐tests, F for ANOVA and H for Kruskal–Wallis).

## 3 Results

### 3.1 Design and Characterization of GDQM‐Loaded NEs

**FIGURE 1:**
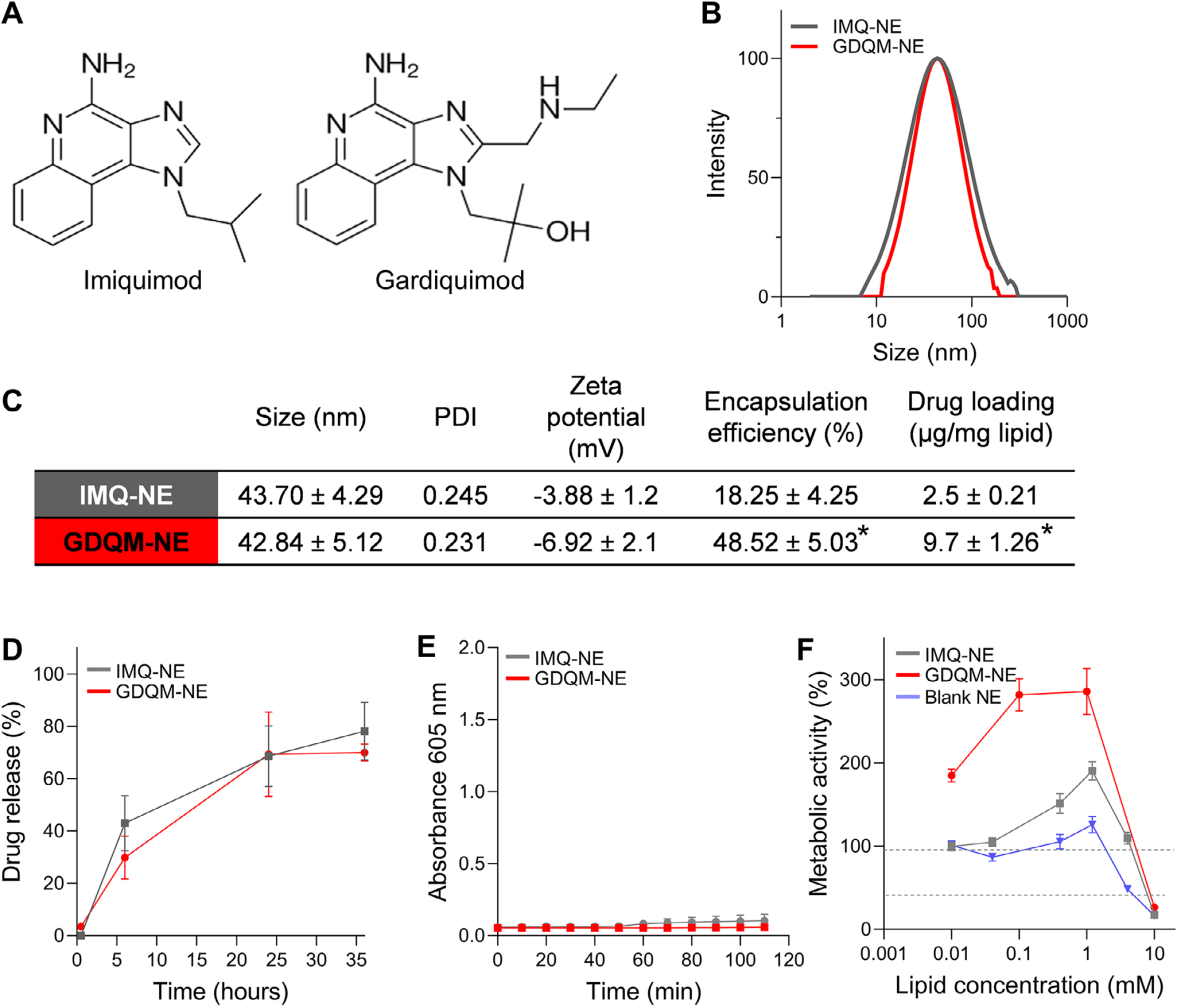
Characterization of IMQ‐ and GDQM‐loaded NEs. (A) Chemical structures of IMQ and GDQM. (B) Representative particle size distribution profiles determined by DLS. (C) Summary of key physicochemical parameters, including mean particle size, PDI, zeta potential, drug loading, and encapsulation efficiency. (D) In vitro release profiles of IMQ and GDQM from NEs in 10% FBS at 37°C and pH 7.4 (*n* = 3). (E) Colloidal stability of NEs in biological medium (DMEM), assessed by turbidity measurements as a surrogate for aggregation. (F) Cytotoxicity of IMQ, GDQM, and blank‐NEs in BMDMs, evaluated by MTT assay. Data are presented as mean ± SD (*n* = 6 in B, C; *n* = 3 in D; and *n* = 12 in E,F). *p*‐values were calculated using unpaired two‐tailed Welch's t‐tests. Among the physicochemical parameters analyzed in C, only encapsulation efficiency was significantly different (*t* = 5.33). *p*‐values in F were calculated by comparing fitted IC_50_ values obtained from nonlinear regression analysis of concentration–response curves. No statistically significant differences were observed in D, E, or F. Asterisks indicate statistically significant differences between the indicated groups: ^*^ *p* < 0.05, ^**^ *p* < 0.01, ^***^ *p* < 0.001, ^****^ *p* < 0.0001.

In order to generate a targeted delivery platform for GDQM, the compound was encapsulated into a NE previously optimized in our laboratory for IMQ [26], composed of OA, Suppocire CM, PEG40‐stearate, and lecithin. Given the structural and physicochemical similarities between both TLR7 agonists (Figure 1A), the same formulation strategy was applied. GDQM‐NEs preserved the physicochemical profile of the IMQ‐loaded NE, displaying comparable hydrodynamic size and PDI values (42.84 ± 5.12 nm vs 43.70 ± 4.29 nm, respectively) (Figure 1B,C), as well as a similar surface charge (−6.92 ± 2.10 mV vs −3.88 ± 1.20 mV). However, GDQM‐NEs showed markedly higher encapsulation efficiency (48.85 ± 5.03% vs 18.25 ± 4.25%) and drug loading (9.7 ± 1.26 vs 2.5 ± 0.21 µg/mg lipid), representing a substantial improvement over IMQ‐NEs. This improvement is consistent with the higher solubility of GDQM in OA compared to IMQ (220 mg/mL versus 121 mg/mL [26], nearly twofold), facilitating its incorporation into the lipid matrix. Although direct comparisons remain challenging due to variability in reporting and formulation strategies, drug loading values for other imidazoquinoline‐based systems (e.g., R848 or IMQ) typically range between ∼5–10% (w/w) in liposomal formulations [31, 32]. In our system, GDQM‐NEs achieved a drug loading of ∼1% (w/w), which, while lower, is sufficient for systemic administration in our experimental setting and enables the delivery of therapeutically relevant doses. Importantly, this is achieved without the need for chemical modification or conjugation of the agonist [33], preserving its native structure and simplifying formulation. Moreover, the selected formulation maintains a small particle size, colloidal stability, and controlled release, all of which are critical parameters for in vivo performance and not always simultaneously achieved in alternative delivery systems [34].

The in vitro release profiles of GDQM and IMQ from the NEs were comparable, with 69.37 ± 16.18% and 68.64 ± 11.62% release at 24 h, and 69.96 ± 3.21% and 78.13 ± 11.05% at 36 h, respectively (Figure 1D), confirming a controlled‐release behavior. The formulation also exhibited good stability in biological media, with no increase in turbidity or signs of aggregation during the 2 h incubation (Figure 1E). Cytotoxicity in BMDMs was comparable between GDQM‐NEs and IMQ‐NEs, with IC_50_ values of 7.22 ± 1.24 mm and 6.56 ± 0.76 mm, respectively (Figure 1F). Notably, at low concentrations, GDQM‐NEs induced a metabolic activity above baseline, consistent with their stronger immunostimulatory potential. Blank‐NE displayed a similar toxicity profile to the drug‐loaded formulations, indicating that cytotoxicity is primarily driven by the nanocarrier structure/concentration rather than the encapsulated agonist. SEM imaging further confirmed that GDQM‐NEs consist of spherical particles with diameters between 40 and 70 nm (Figure S1).

### 3.2 In Vitro Cellular Uptake and Intracellular Localization of DiD‐Labeled NE by BMDM

**FIGURE 2:**
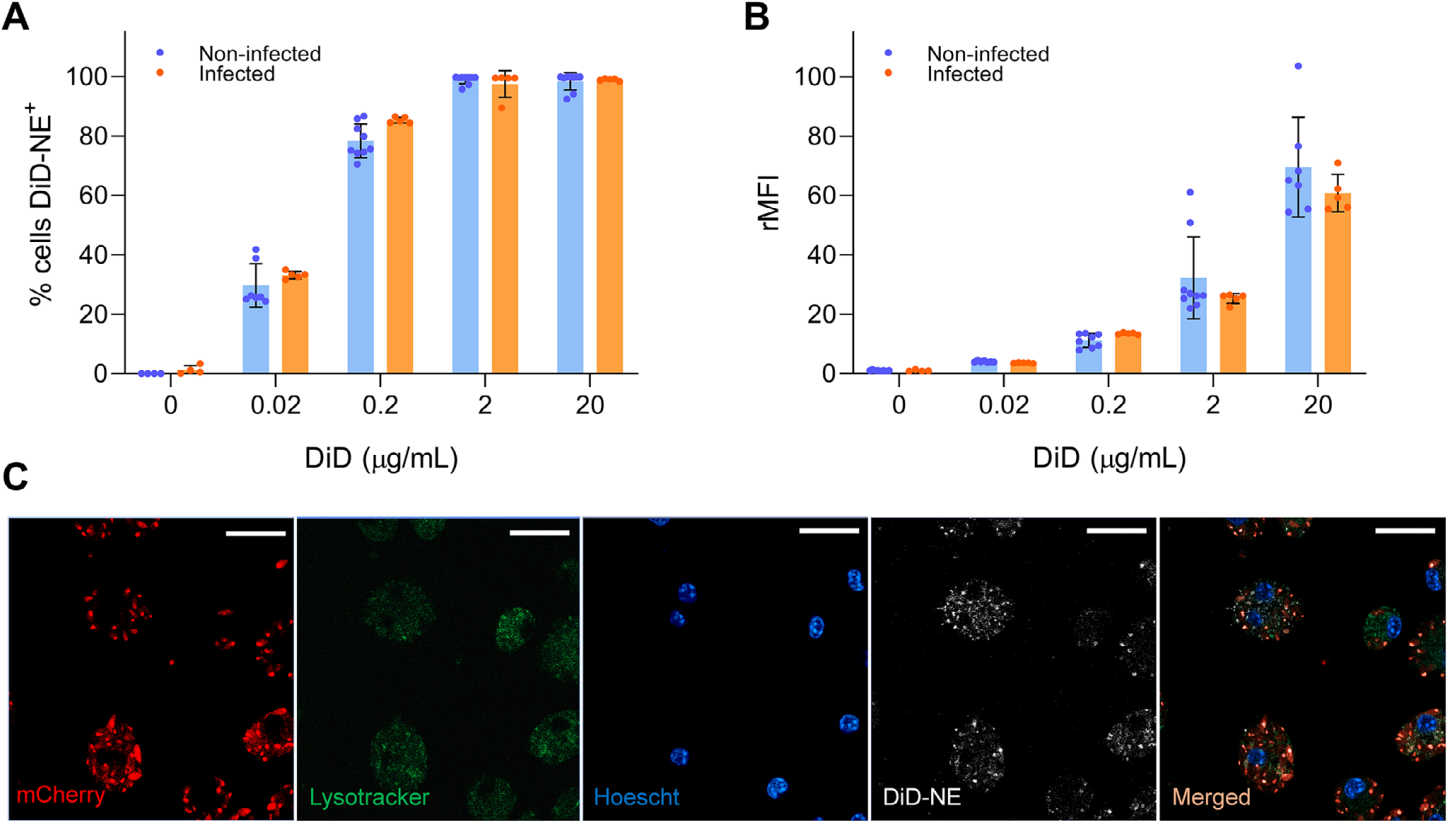
Uptake of GDQM‐loaded NEs by BMDMs in vitro. BMDMs, infected or not with *L. major*, were incubated with different concentrations of DiD‐labeled NEs (1% w/w) for 3 h. (A) Percentage of DiD‐positive cells. (B) rMFI of DiD‐positive cells. Data represent two independent experiments (*n* = 6–12). *p‐*values were calculated using two‐way ANOVA followed by Šídák's multiple comparisons test to compare uninfected and *L. major*‐infected BMDMs within each NE concentration (*F* = 0.27 for A and 0.76). No statistically significant differences were observed. (C) Representative 63x fluorescence images of BMDMs infected with *L. major*‐mCherry (red), lysosomes labeled with LysoTracker (green), and nuclei stained with Hoechst (blue). DiD signal is shown in white, indicating NEs localization within macrophages. Scale bar: 20 µm.

Uptake of GDQM‐loaded NEs was assessed in vitro over 3 h using DiD‐labeled NEs in both non‐infected and *L. major*–infected BMDMs to determine whether *Leishmania* infection affects particle internalization. Although some studies report that *Leishmania* infection can affect phagocytosis of different types of nanoparticles macrophages [35, 36, 37], uptake of this specific type of NEs had not been previously examined. As shown in Figure 2A,B, GDQM‐NEs were efficiently and rapidly internalized under all conditions, and no significant differences were observed between infected and non‐infected BMDMs, indicating that infection does not measurably modify the uptake of these formulations. Uptake approached saturation in terms of DiD‐positive cells at concentrations ≥ 2 µg/mL, where almost the entire macrophage population became NE‐positive. Nevertheless, the rMFI continued to increase at 20 µg/mL, indicating that uptake capacity had not yet reached its maximal threshold. This was further supported by the increase in cellular complexity (SSC) observed at higher NE concentrations (Figure S2).

Beyond quantifying uptake, it is essential to assess whether the internalized NEs reach the subcellular compartments necessary for target activation. In this case, TLR7 is an endosomal receptor, so colocalization with endolysosomal compartments is critical. Fluorescence microscopy using LysoTracker to label lysosomes, mCherry‐expressing *L. major*, and DiD‐labeled NEs demonstrated a significant overlap between the lysosomal marker, the parasite, and the NEs (Figure 2C). This in vitro analysis establishes that GDQM‐NEs are efficiently internalized and correctly trafficked to the endosomal compartment, laying the groundwork for subsequent in vivo studies of parasite‐drug colocalization in infected tissues.

### 3.3 GDQM‐NEs Promote a Pro‐Inflammatory Macrophage Phenotype at the Transcriptional and Surface Marker Levels in Both Non‐Infected and Infected BMDM

**FIGURE 3:**
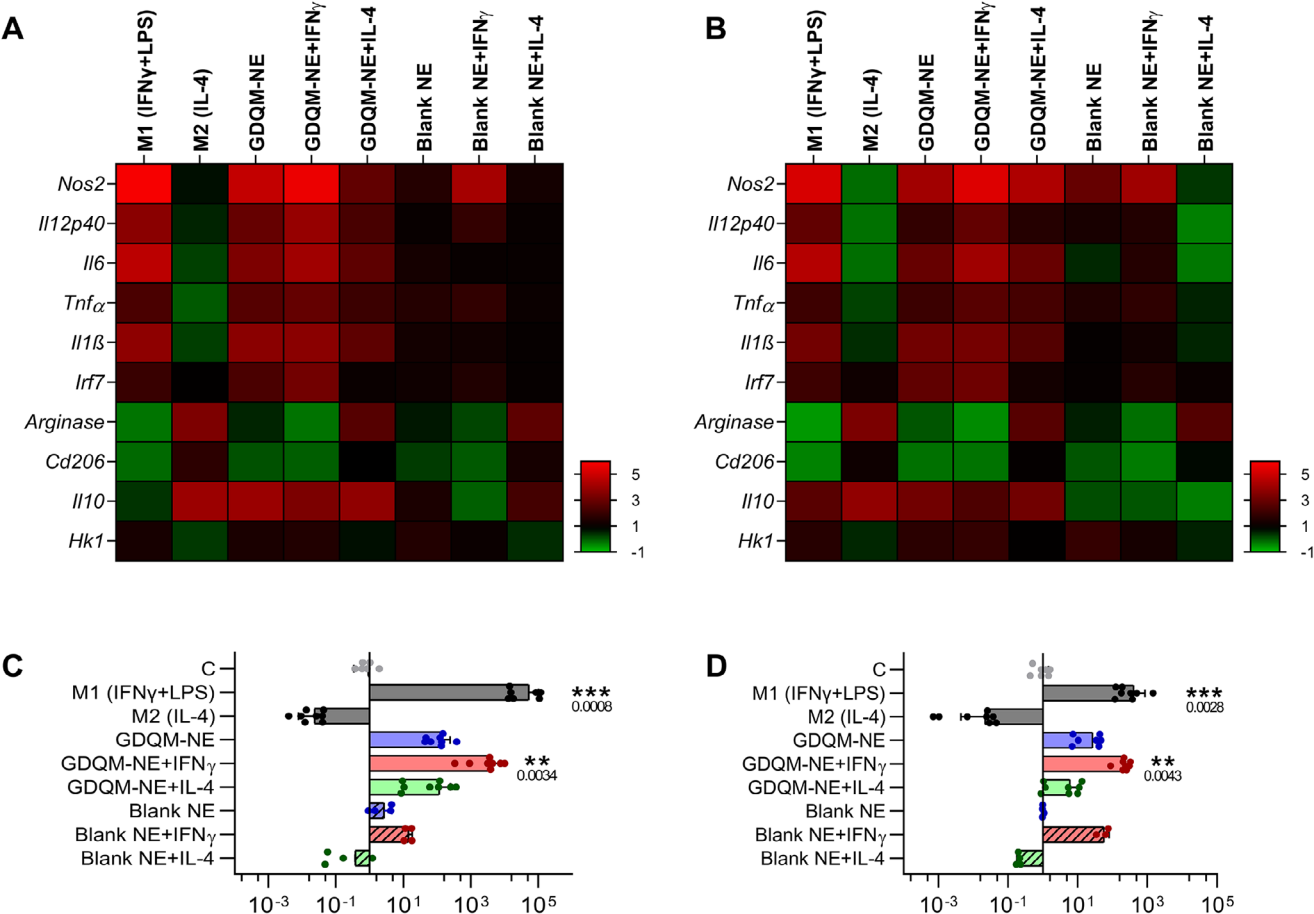
RT‐qPCR analysis of gene expression in uninfected and *Leishmania major*–infected BMDMs treated for 6 h with GDQM‐NEs (1 µm GDQM) or blank NEs (50 µm lipids) under different co‐stimulation conditions. Left panels (A,C) correspond to uninfected BMDM, whereas right panels (B,D) correspond to BMDM infected with *L. major*. (A,B) Log_10_ fold‐change heat maps showing the expression profiles of a panel of ten macrophage polarization–related genes quantified by RT‐qPCR. Each square represents the mean Log_10_ fold‐change from two independent experiments, calculated from ΔCt values relative to control samples. Green indicates increased expression, whereas red indicates decreased expression. (C,D) Ratios between M1‐associated genes (*Il6, Nos2, Tnfa, Il12p40*, and *Il1b*) and *M2*‐associated genes (*Arg1, Il10*, and *Cd206*). Data are presented as mean ± SD of two independent experiments (*n* = 6–12). *p*‐values were calculated using the Kruskal–Wallis test followed by Dunn's multiple comparisons test; *H* = 53.27 for panel C and *H* = 52.41 for panel D. Asterisks indicate statistically significant differences relative to the corresponding untreated control: ^*^ *p* < 0.05, ^**^ *p* < 0.01, ^***^ *p* < 0.001, ^****^ *p* < 0.0001.

**FIGURE 4:**
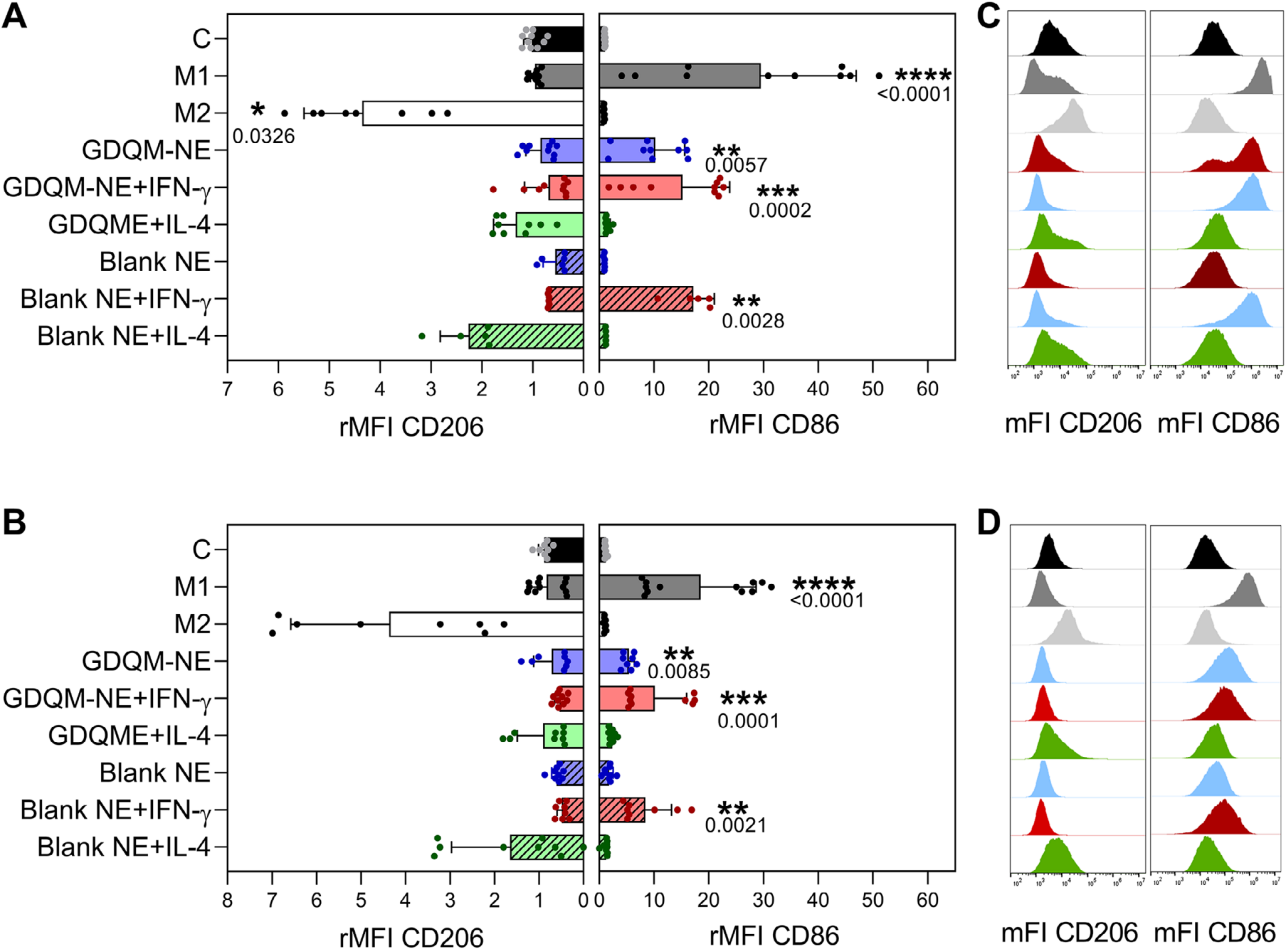
Immunophenotyping by flow cytometry in BMDMs treated with GDQM‐NE or blank formulations for 48 h. BMDMs were treated for 48 h with 10 µm GDQM‐NEs or blank NEs containing an equivalent lipid concentration (500 µm). Upper panels (A–C) correspond to uninfected macrophages, and lower panels (B–D) to *L. major*‐infected macrophages. (A,B) rMFI of polarization markers CD206 and CD86, and (C,D) representative histograms for each condition. Data are presented as mean ± SD of two independent experiments (*n* = 5–12). *p*‐values were calculated using the Kruskal–Wallis test followed by Dunn's multiple comparisons test relative to the corresponding untreated control; *H* = 69.49 for panel A and *H* = 33.03 for panel B. Comparisons between infected and uninfected macrophages within the same treatment groups were also performed and were not statistically significant. Asterisks indicate statistically significant differences relative to the corresponding untreated control: ^*^ *p* < 0.05, ^**^ *p* < 0.01, ^***^ *p* < 0.001, ^****^ *p* < 0.0001.

To evaluate the immunomodulatory capacity of both GDQM‐loaded and blank NEs, we first analyzed the expression profile (Figure 3) and the surface marker levels (Figure 4) of BMDM exposed to the formulations. BMDMs were treated under conditions that included co‐stimulatory cytokines (IFN‐γ as a pro‐M1 signal or IL‐4 as a pro‐M2 signal) to assess their effects in more complex immunological contexts. Blank NEs were included to distinguish the intrinsic contribution of the lipid carrier, as lipids themselves can exert immunostimulatory effects beyond their structural or energetic roles. Experiments were performed in both uninfected and *Leishmania major*–infected macrophages. In preliminary experiments, the corresponding free agonist (GDQM) was also included. However, no significant differences were observed between free and nanoencapsulated GDQM in terms of gene expression (*data not shown*). This is consistent with previous observations in macrophage systems, where small‐molecule immunomodulators can readily access intracellular targets by diffusion under simplified in vitro conditions [38], resulting in comparable responses regardless of the delivery format.

RT‐qPCR analysis was conducted after 6 h of treatment with GDQM‐NEs (1 µM GDQM) or lipid‐equivalent blank NEs (50 µm). This concentration was selected based on preliminary dose–response experiments, in which lower concentrations did not induce detectable changes in gene expression, whereas higher concentrations resulted in similar activation profiles, indicating a plateau from 1 µm onward (*data not shown*). Heatmaps (Figure 3A,C) show relative expression of ten macrophage polarization–related genes compared to untreated controls (red: upregulated; black: unchanged; green: downregulated). GDQM‐NEs markedly enhanced the transcription of key M1‐associated and pro‐inflammatory genes, including *Nos2, Il12p40, Il6, Tnfa*, and *Il1b*, reaching levels comparable to those observed in stimulated M1 controls (IFN‐γ+LPS). Notably, *Irf7*, a transcription factor directly linked to TLR7 signaling [39], was also strongly upregulated, suggesting activation of pathways associated with the drug's molecular target. In parallel, genes involved in glycolytic metabolism such as *Hk1* were increased, consistent with the metabolic reprogramming typical of classically activated macrophages. Conversely, M2‐related markers such as *Arg1* and *Cd206* were significantly downregulated, reinforcing the pro‐inflammatory bias induced by GDQM‐NEs. Blank‐NEs also promoted a moderate induction of M1 genes, though to a lesser extent, indicating that the lipid carrier exerts a mild immunostimulatory effect. These trends are further reflected in the M1/M2 gene expression ratios (Figure 3C,D), which clearly show a pronounced M1 polarization in GDQM‐NE–treated macrophages. In *L. major*–infected macrophages, a similar transcriptional pattern was observed, albeit with slightly attenuated M1/M2 ratios, consistent with the parasite's known ability to dampen inflammatory responses. Interestingly, GDQM‐NEs also induced *Il10* expression, which was absent in blank NEs, suggesting a feedback mechanism to modulate inflammation, as previously observed with our IMQ‐loaded NEs and other types of TLR agonists [26, 40].

At the protein level, after 48 h of exposure, macrophages were analyzed by flow cytometry for surface markers CD86 (M1) and CD206 (M2) (Figure 4). GDQM‐NE treatment significantly increased CD86 expression and reduced CD206, particularly in combination with IFN‐γ, in both uninfected (Figure 4A,B) and infected macrophages (Figure 4C,D). This effect highlights the strong capacity of GDQM‐NEs to promote classical macrophage activation at the protein level, in agreement with the transcriptional profiles observed in RT‐qPCR. Co‐stimulation with IL‐4 did not increase CD86 expression; however, CD206 levels remained lower than in IL‐4 controls, indicating that GDQM‐NEs can partially counteract M2‐skewing conditions. This M1‐oriented response‐characterized by increased CD86 and decreased CD206‐was slightly attenuated in *L. major*–infected macrophages, although the difference between infected and uninfected groups was not statistically significant. Blank NEs induced only modest CD86 upregulation on their own, but CD86 levels increased markedly when combined with IFN‐γ, reaching values similar to GDQM‐NEs under the same cytokine conditions. Under IL‐4 co‐stimulation, blank NEs produced intermediate CD206 levels ‐lower than in M2 controls but higher than in the other treatments‐ with no significant differences between infected and uninfected macrophages. This pattern overall highlights the dominant influence of cytokine co‐stimulation on macrophage activation.

### 3.4 GDQM‐NEs Reinforce Macrophage Activation at the Functional Level: Cytokine, ROS, and NO Production

**FIGURE 5:**
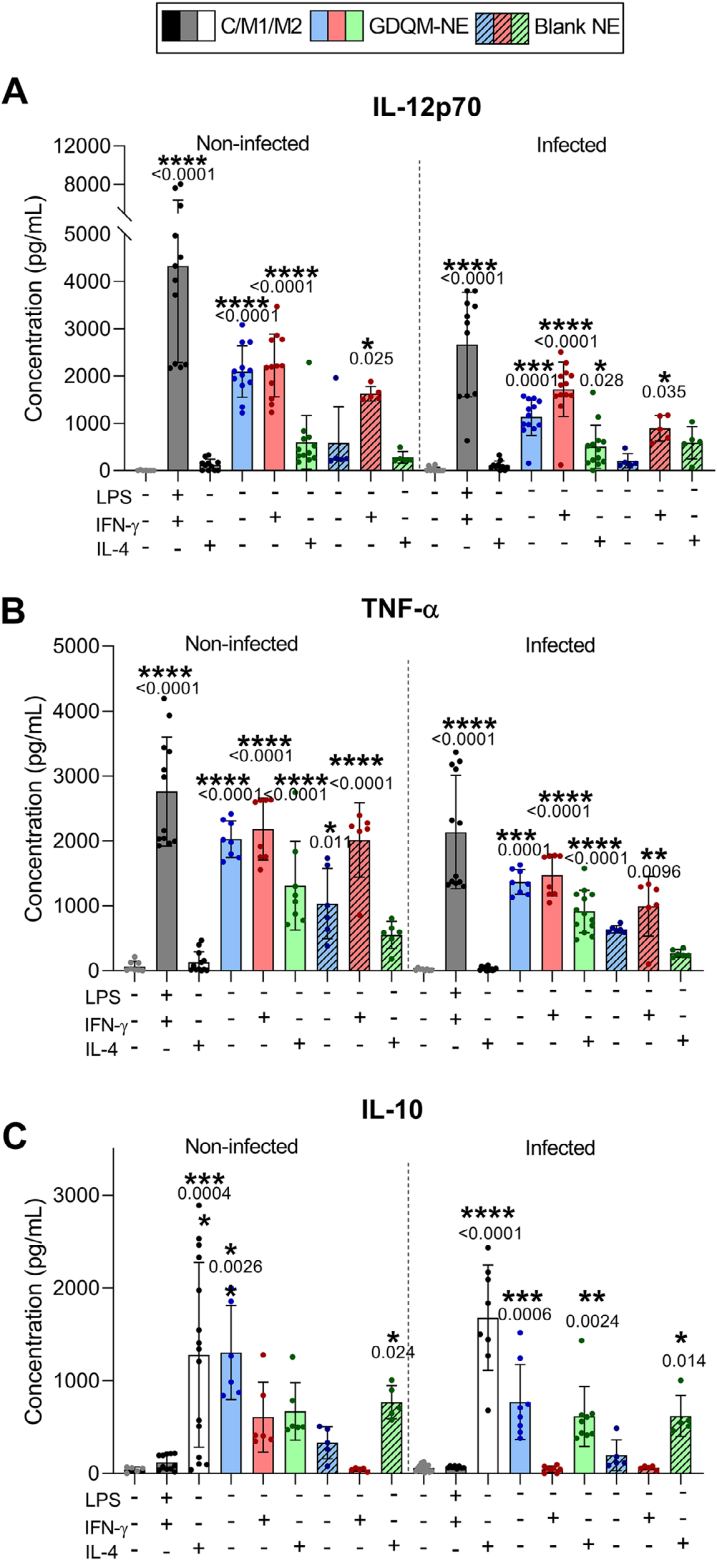
Cytokine production by BMDMs treated with GDQM‐NE or blank formulations. BMDMs were treated for 48 h with 10 µm GDQM‐NEs or blank NEs containing an equivalent lipid concentration (500 µm). Panels show cytokine levels in culture supernatants quantified by ELISA. (A) IL‐12p70; (B) TNF‐α; (C) IL‐10. Data are presented as mean ± SD of two independent experiments (*n* = 6–12). *p*‐values were calculated using the Kruskal–Wallis test followed by Dunn's multiple comparisons test relative to the corresponding untreated control; *H* = 140.4 for panel A, *H* = 132.4 for panel B, and H = 101.9 for panel C. Comparisons between infected and uninfected macrophages within the same treatment groups were also performed and were not statistically significant. Asterisks indicate statistically significant differences relative to the corresponding untreated control: ^*^ *p* < 0.05, **p < 0.01, ^***^ *p* < 0.001, ^****^ *p* < 0.0001.

**FIGURE 6:**
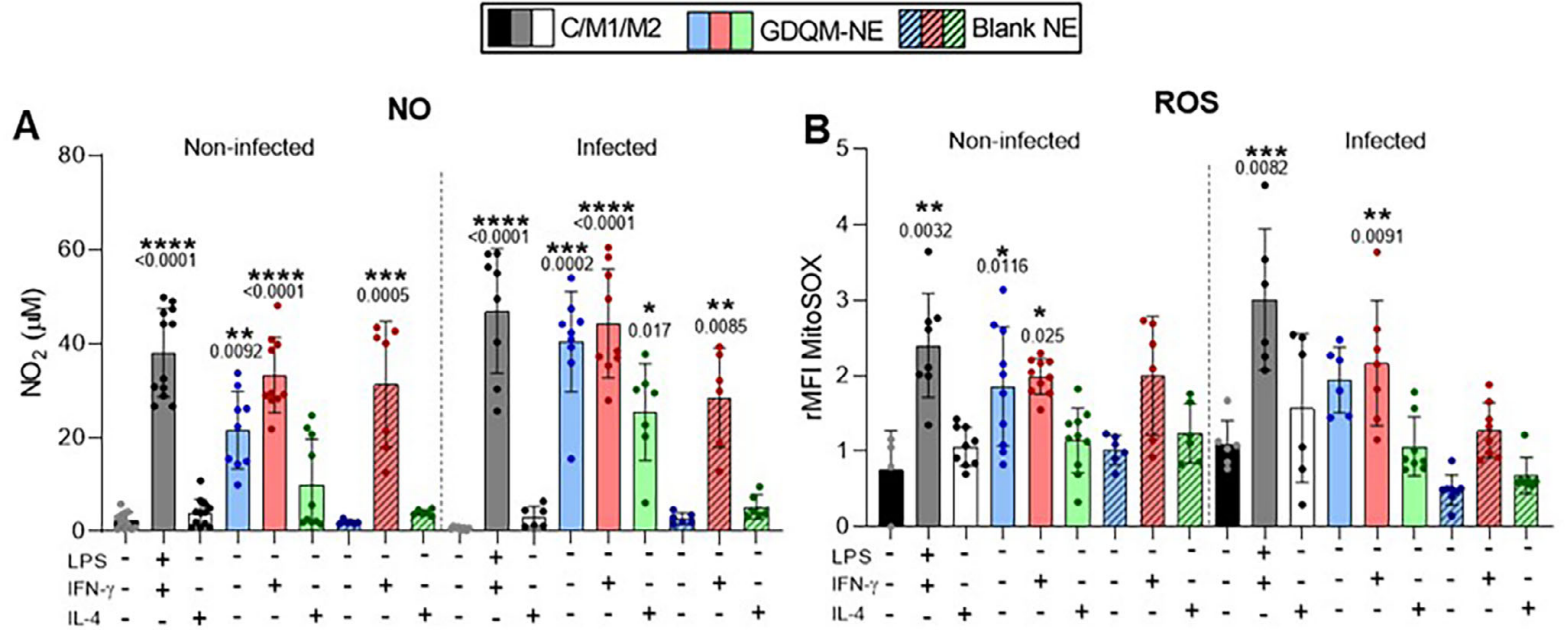
NO and ROS production by BMDMs treated with GDQM‐NE or blank formulations. BMDMs were treated for 48 h with 10 µm GDQM‐NEs or blank NEs containing an equivalent lipid concentration (500 µm). Panels show NO (A) and ROS (B) levels in culture supernatants or cells, combining uninfected and *L. major*‐infected macrophages. NO was measured by the Griess assay, and ROS by flow cytometry using MitoSOX in viable F4/80^+^ MitoTracker^+^ macrophages, expressed as rMFI relative to untreated controls. Data are presented as mean ± SD from two independent experiments (*n* = 6–12). *p*‐values were calculated using the Kruskal–Wallis test followed by Dunn's multiple comparisons test relative to the corresponding untreated control; *H* = 122.1 for panel A and *H* = 81.02 for panel B. No significant differences were observed between infected and uninfected cells within the same treatments. Asterisks indicate statistically significant differences relative to the corresponding untreated control: ^*^ *p* < 0.05, ^**^ *p* < 0.01, ^***^ *p* < 0.001, ^****^ *p* < 0.0001.

To further characterize the functional phenotype induced by GDQM‐NEs, we analyzed the production of key inflammatory mediators and effector molecules in the supernatants of treated BMDMs after 48 h (Figures 5 and 6). The same experimental setup used in the transcriptional and surface marker assays was applied here, including the presence or absence of IFN‐γ (pro‐M1) or IL‐4 (pro‐M2) co‐stimulation. Both uninfected and *L. major*–infected macrophages were evaluated. The 48 h time point was selected based on preliminary time‐course experiments, which showed no detectable production of effector molecules at early time points and maximal responses at 48 h (*data not shown*). In addition, dose–response analyses indicated that effector molecule production increased with GDQM concentration and reached maximal levels at 10 µM (*data not shown*), supporting the selection of these conditions for functional characterization.

Cytokine analysis focused on TNF‐α, IL‐12p70, and IL‐10 secretion (Figure 5A–C). In line with the strong M1‐like transcriptional and phenotypic signatures described above, GDQM‐NE treatment led to a robust and significant increase in TNF‐α and IL‐12p70 levels compared with untreated controls. This effect was further amplified when GDQM‐NEs were combined with IFN‐γ; under this condition, TNF‐α and IL‐12p70 levels were comparable between GDQM‐NEs and blank NEs, indicating that IFN‐γ co‐stimulation dominates the pro‐inflammatory response regardless of nanoparticle cargo. TNF‐α secretion remained significantly elevated even under IL‐4 co‐stimulation, consistent with the ability of GDQM‐NEs to partially counteract M2‐polarizing signals. These trends were consistent in both infected and uninfected BMDMs. Notably, GDQM‐NEs also induced significant IL‐10 production (Figure 5C), mirroring the transcriptional upregulation of *Il10* observed by RT‐qPCR, which likely reflects a regulatory feedback mechanism that prevents excessive inflammation. In contrast, blank NEs triggered only modest cytokine secretion, with TNF‐α and IL‐12p70 increases observed exclusively under IFN‐γ co‐stimulation, and IL‐10 detected at significant levels mainly in the presence of IL‐4.

Given the strong induction of *Nos2* expression detected in previous assays, NO production was next quantified using the Griess reaction (Figure 6A). GDQM‐NEs markedly enhanced NO release, reaching levels comparable to M1 controls, particularly in the presence of IFN‐γ. Blank NEs elicited only minimal NO production, consistent with their weaker immunostimulatory profile. Finally, intracellular ROS generation was evaluated by flow cytometry (Figure 6B). Importantly, the only treatments that promoted a significant increase in ROS levels were those involving GDQM‐NEs—either alone or in combination with IFN‐γ—while blank NEs failed to elicit detectable ROS induction. ROS production remained elevated, though attenuated, in infected macrophages. The combined upregulation of NO and ROS underscores the strong activation of macrophage microbicidal functions induced by GDQM‐NEs, in line with their potent pro‐inflammatory and TLR7‐dependent signaling activity.

### 3.5 GDQM‐NEs and Blank‐NEs Drive a Robust Metabolic Reprogramming Toward Aerobic Glycolysis and Reduced Mitochondrial Respiration

**FIGURE 7:**
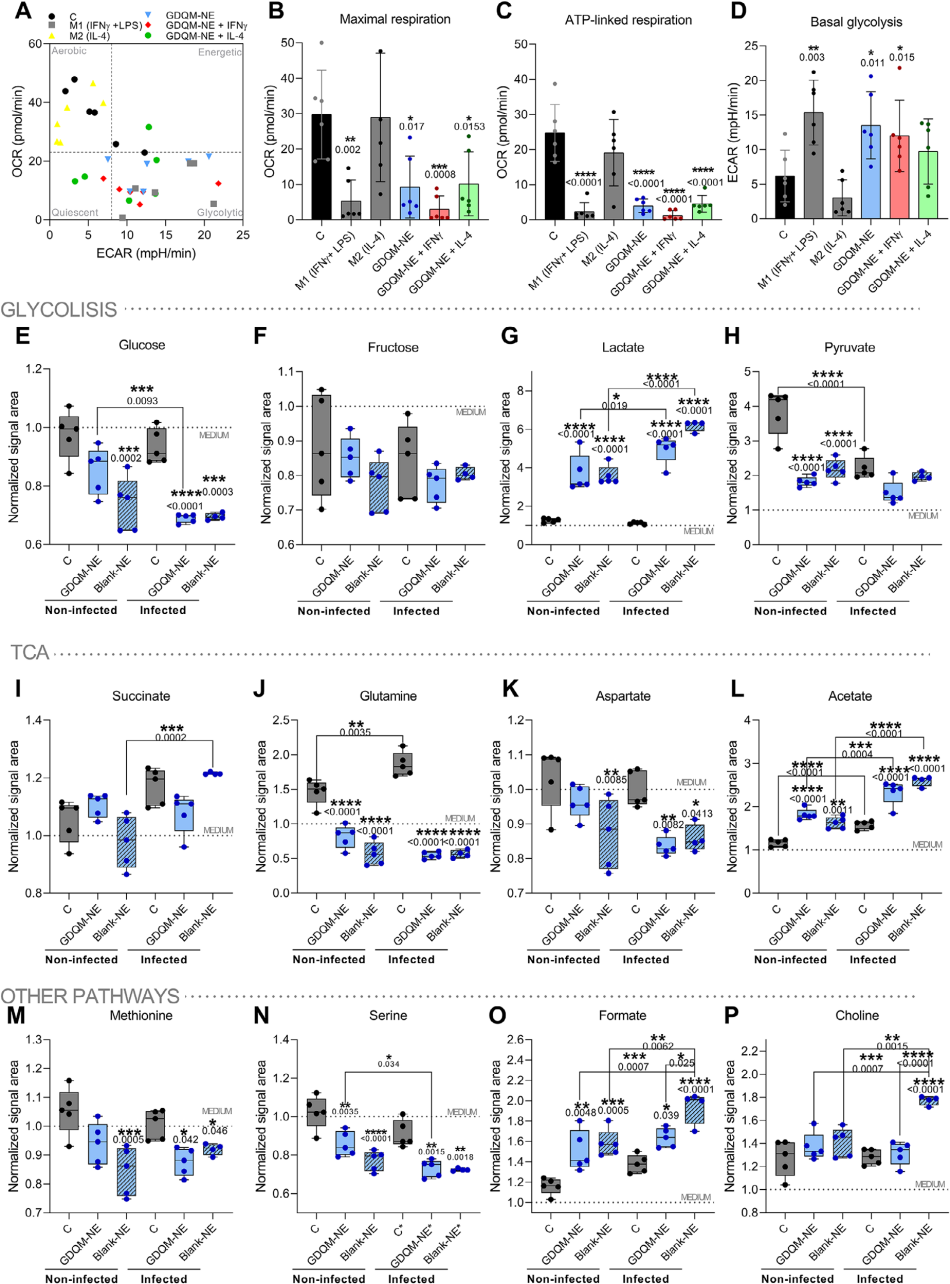
Metabolic profile induced by GDQM‐NE and blank NE in BMDMs after 48 h treatment. BMDMs were treated for 48 h with 10 µm GDQM‐NEs or blank NEs containing an equivalent lipid concentration (500 µm lipids). (A–D): Seahorse analysis of uninfected BMDM. (A) Dotplot of basal respiration versus basal glycolysis; (B) maximal respiration; (C) ATP‐linked respiration; (D) basal glycolysis. Data are presented as mean ± SD of two independent experiments (*n* = 6). E–P: NMR‐based metabolomic analysis of culture medium supernatants from uninfected and *L. major*‐infected macrophages: metabolite signal areas in cell‐conditioned media normalized to acellular medium (represented by the dashed line). (E–H) Glycolysis‐related metabolites: glucose (E), fructose (F), lactate (G), pyruvate (H). (I–L) TCA cycle metabolites: succinate (I), glutamine (J), aspartate (K), acetate (L). (M–O) one‐carbon metabolism and related metabolites: methionine (M), serine (N), formate (O). (P) Choline. Data are presented as mean ± SD of one experiment (n = 5). *p*‐values were calculated using one‐way ANOVA followed by Tukey's multiple comparisons test relative to untreated controls. Test statistics were as follows: F = 32.81 for B, F = 57.32 for C, F = 24.64 for D, F = 16.48 for E, F = 1.23 for F, F = 60.06 for G, F = 24.91 for H, F = 7.846 for I, F = 70.92 for J, F = 7.632 for K, F = 52.84 for L, F = 7.308 for M, F = 17.66 for N, F = 25.43 for O, and F = 12.20 for P. Asterisks above columns indicate statistically significant differences relative to the corresponding untreated control: ^*^ *p* < 0.05, ^**^ *p* < 0.01, ^***^ *p* < 0.001, ^****^ *p* < 0.0001. Comparisons between infected and uninfected macrophages within the same treatment are indicated by connecting lines where appropriate.

Building on the transcriptional, phenotypic, and functional analyses, we next investigated whether our NEs also reprogram macrophage metabolism. Cellular metabolism not only sustains macrophage activity and polarization but also acts as an active regulator of immune function [41]. Thus, we examined how the formulations (GDQM‐NEs and Blank‐NEs) reshape BMDM bioenergetics and extracellular metabolite fluxes (Figure 7), which are intimately linked to inflammatory and microbicidal capacities.

We first performed a Seahorse extracellular flux analysis using GDQM‐loaded NEs (Figure 7 and Figure S3), assessing OCR and ECAR as indicators of mitochondrial respiration and glycolytic flux, respectively. Plotting basal ECAR against basal OCR revealed four metabolic phenotypes (Figure 7A), allowing us to position each treatment group within established metabolic archetypes. As expected, M2 macrophages displayed a respiration‐dominated profile (aerobic metabolism), relying primarily on mitochondrial metabolism [41]. In contrast, both M1 controls and GDQM‐NE–treated macrophages clustered in the low‐OCR/high‐ECAR quadrant, indicative of a strongly glycolytic phenotype, consistent with the metabolic hallmark of classically activated macrophages described extensively in the literature [41]. Consistently, GDQM‐NEs markedly reduced maximal respiration (Figure 7B) and ATP‐linked OCR (Figure 7C) relative to untreated BMDM and IL‐4–treated M2 controls. This reduction in mitochondrial function mirrors the well‐known breakpoints in the Tricarboxylic acid (TCA) cycle occurring during pro‐inflammatory polarization.

Given the prominent metabolic shifts observed by Seahorse, we next performed NMR metabolomic analysis of macrophage supernatants to characterize changes in extracellular metabolite consumption and secretion (Figure 7E–O). Both GDQM‐NEs and blank NEs were analyzed in infected and uninfected BMDMs. Baseline levels were determined from acellular medium controls, and variations relative to this reference were taken to reflect net secretion or consumption by the cells.

Focusing on the metabolic pathway most strongly associated with M1‐macrophage polarization, glycolysis (Figure 7E–H), we detected a clear shift toward a glycolytic phenotype across all NE‐treated conditions, similar to Seahorse previous data. Both glucose and fructose were markedly consumed, while lactate accumulated in the extracellular medium, accompanied by reduced pyruvate levels, indicating rapid conversion of pyruvate into lactate. This pattern reflects an enhanced glycolytic flux following both GDQM‐NE and blank NE treatment.

Within TCA‐related metabolites (Figure 7I–L), succinate levels increased modestly but remained close to untreated controls indicating that although TLR activation can enhance intracellular succinate accumulation, its release to the extracellular space is limited under these conditions. In contrast, glutamine was consistently consumed in all NE conditions, whereas untreated macrophages displayed net glutamine release. Elevated glutamine consumption aligns with an M1‐like phenotype, where glutaminolysis feeds a truncated TCA cycle [41]. Indeed, glutamine metabolism and uptake dynamics are known to be modulated activated by TLR activation [42], and by exposure to saturated fatty acids [43]. Aspartate (Figure 7K) levels also decreased across treatments, further supporting increased anaplerotic flux to sustain nucleotides and aminoacids synthesis [44]. Acetate (Figure 7L) secretion increased in all treatments, particularly in infected macrophages, likely reflecting enhanced glycolysis and catabolism of internalized lipids due to lipid overload.

Methionine (Figure 7M) and serine (Figure 7N) both showed significantly decreased levels in the supernatants of all treated conditions, indicating increased utilization. Serine catabolism to glycine via the one‐carbon (1C) pathway generates one‐carbon units, which are largely released as formate (Figure 7O), consistent with activation of this metabolic route. This pathway is directly linked to macrophage polarization: low serine and active methionine utilization support the production of S‐adenosylmethionine (SAM), the universal methyl donor required for epigenetic reprogramming of inflammatory genes and for promoting a proinflammatory state [45, 46]. The observed decreases in both serine and methionine further underscore the engagement of this metabolic‐epigenetic axis in NE‐stimulated macrophages

Another interesting metabolite is choline, a quaternary amine and precursor of PC, which is one of the lipid components of the NEs used in this study. In M1 macrophages, characterized by increased lipid synthesis, PC has been shown to be important for the secretion of proinflammatory cytokines [47]. PC is also the most abundant lipid in *Leishmania*, and its uptake is critical for its survival [48]. Thus, both a proinflammatory phenotype and the presence of the parasite create a high demand for choline; however, the exogenous PC overload provided by the NE may limit further uptake, as shown in Figure 7P. Interestingly, choline accumulation was observed in infected macrophages treated with blank NE, likely because M1‐like polarization is less intense (as observed in previous sections) and the parasite's demand is limited, reducing intracellular consumption and favoring its release into the supernatant.

### 3.6 GDQM‐NEs Enhance Macrophage Anti‐*Leishmania* Activity

**FIGURE 8:**
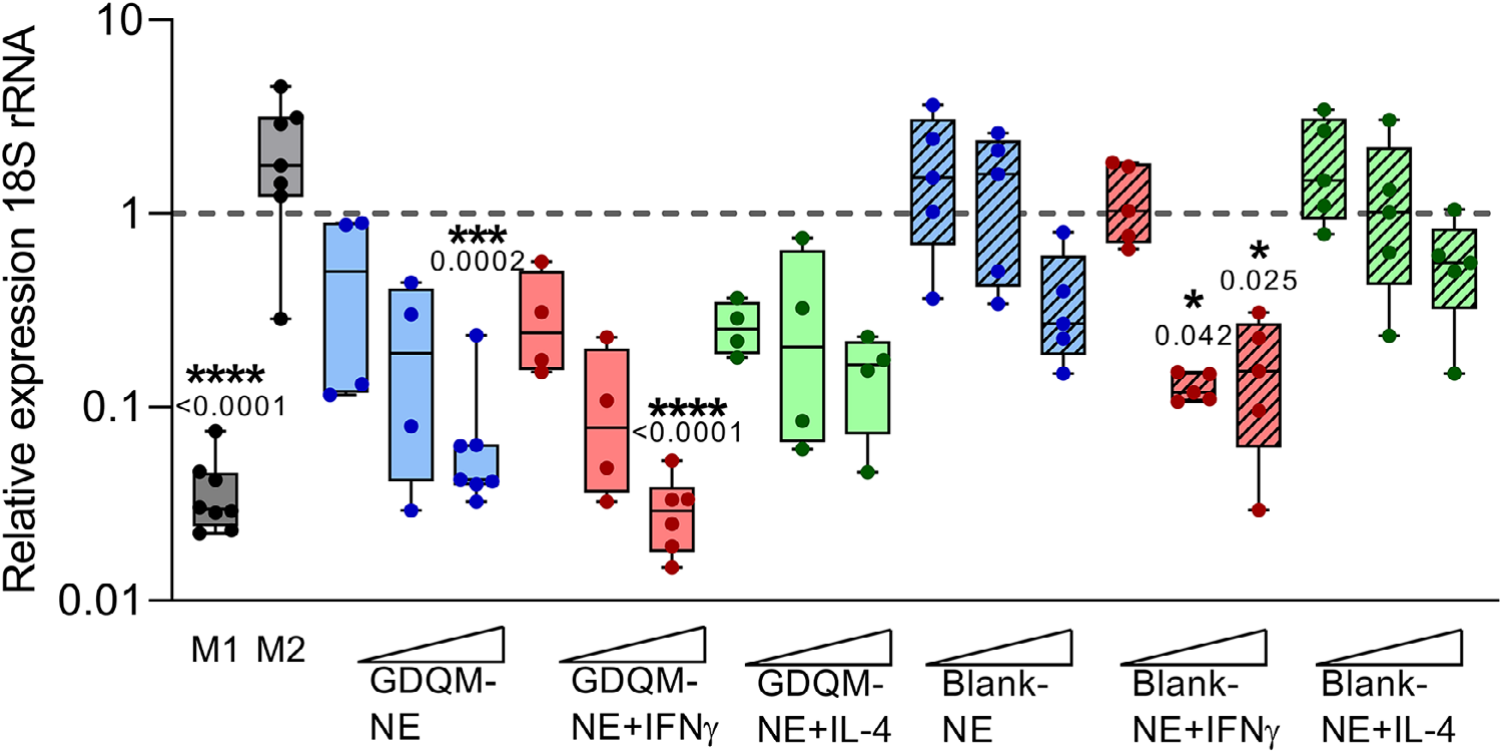
In vitro quantification of intracellular *Leishmania* load in BMDMs following treatment with GDQM‐loaded or blank NEs with co‐stimuli. Parasite burden was assessed by RT‐qPCR measuring *Leishmania* 18S rRNA after 48 h of treatment. BMDMs were treated with GDQM‐NEs at 0.1, 1, and 10 µm GDQM (indicated by increasing triangles) or with blank NEs at equivalent lipid concentrations (5, 50, and 500 µm lipids, respectively). Data are presented as mean ± SD from two independent experiments (*n* = 4–8). *p*‐values were calculated using the Kruskal–Wallis test followed by Dunn's multiple comparisons test relative to untreated controls; H = 78.83. Comparisons between blank and GDQM‐loaded NEs at each concentration and co‐stimulus were also performed and were not statistically significant. Asterisks indicate statistically significant differences relative to the corresponding untreated control: ^*^ *p* < 0.05, ^**^ *p* < 0.01, ^***^ *p* < 0.001, ^****^ *p* < 0.0001.

Given the robust pro‐inflammatory and M1‐skewing effects induced by GDQM‐NEs across multiple cellular layers, we next asked whether this activation translated into an improved anti‐*Leishmania* response. To address this, BMDMs were infected with *L. major* for 24 h and subsequently treated for 48 h with GDQM‐NEs at three concentrations (0.1, 1, and 10 µm GDQM), or with lipid‐equivalent blank NEs, under the same co‐stimulation conditions used throughout the study (IFN‐γ or IL‐4). Parasite burden was quantified by qPCR (Figure 8).

As expected, the M1 control (IFN‐γ) showed a strong and significant reduction in parasite load. Notably, GDQM‐NE treatment at 10 µm also led to a marked and significant decrease in parasite burden, both in the absence of co‐stimulation and in combination with IFN‐γ, demonstrating that the potent activation induced by the formulation confers enhanced leishmanicidal capacity to macrophages. Under IL‐4 co‐stimulation, a trend toward parasite reduction was observed, although it did not reach statistical significance, in line with the more permissive nature of M2‐skewed macrophages. Although blank NEs induced partial immunometabolic activation in macrophages, their effect on parasite control was modest. A slight reduction in parasite burden was detected at the highest particle concentration and only in the presence of IFN‐γ, but this decrease was not statistically significant. This pattern parallels their weaker pro‐inflammatory response observed in previous sections.

Collectively, these data indicate that GDQM‐NEs not only reprogram macrophages toward a strongly pro‐inflammatory, glycolytic, and microbicidal phenotype, but also enhance their capacity to control *Leishmania* infection in vitro.

### 3.7 GDQM‐NEs Achieve Co‐Localization With *L. major* in Ears and Sustain Activation of Antigen‐Presenting Cells in dLNs

**FIGURE 9:**
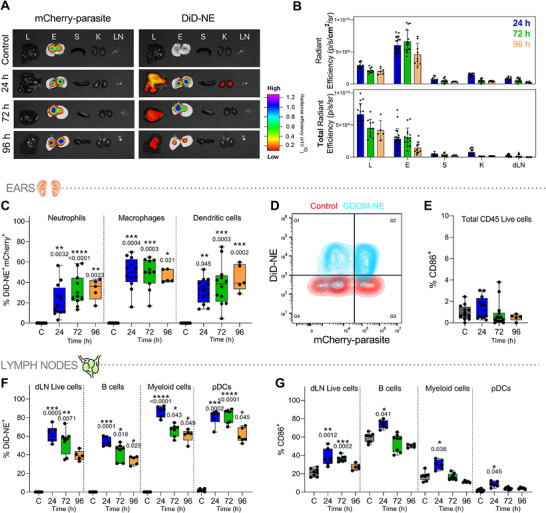
In vivo biodistribution and immune cell uptake of GDQM‐loaded NEs in BALB/c mice with established ear infections of *L. major–*mCherry. BALB/c mice were intradermally infected in the ear with 10^4^ metacyclic promastigotes of *L. major*‐mCherry. Four weeks after infection, mice received a single intravenous dose of DiD‐labeled GDQM‐NEs (1 mg/kg DiD). (A) Ex vivo IVIS imaging of ears, liver, spleen, and kidneys at 24, 48, and 96 h post‐intravenous injection of DiD‐labeled GDQM‐NEs. For each tissue, the left panel shows mCherry fluorescence (*Leishmania* signal) and the right panel shows DiD fluorescence corresponding to GDQM‐NE. (B) Total fluorescence intensity (bottom) and tissue area‐normalized (top) signals show preferential accumulation in infected ears and in the liver. (C) Flow cytometry quantification of double‐positive (DiD^+^ mCherry^+^) myeloid cells in the infected ears (neutrophils, macrophages, dendritic cells), indicating co‐localization of nanoparticles with parasite‐infected cells over time. (D) Representative dot plot of macrophages comparing a control and treated mouse, showing DiD versus mCherry fluorescence; Q2 indicates double‐positive cells reflecting NE uptake by parasite‐infected cells. (E) CD86 expression in ear immune cells, showing absence of activation despite nanoparticle delivery. (F) Flow cytometry of auricular dLNs showing NE uptake by B cells, myeloid cells, and pDCs over time. (G) CD86 expression in dLN cells, demonstrating sustained activation peaking at 24 h. Data are presented as mean ± SD from at least five mice per group across three independent experiments (*n* = 5‐12). *p*‐values were calculated using the Kruskal–Wallis test followed by Dunn's multiple comparisons test relative to untreated controls; H = 83.11 for panel C, H = 5.64 for panel E, H = 60.73 for panel F, and H = 75.24 for panel G. Asterisks above columns indicate statistically significant differences relative to untreated controls: ^*^ *p* < 0.05, ^**^ *p* < 0.01, ^***^ *p* < 0.001, ^****^ *p* < 0.0001.

Due to the challenges of delivering therapeutics to parasite‐infected macrophages in CL, we next assessed the in vivo biodistribution of GDQM‐loaded NEs at 24, 48, and 96 h post‐administration (i.v.) to determine whether they reach the infection site and are internalized by relevant immune cells (Figure 9). For these studies, we used fluorescently labeled GDQM‐NEs containing DiD, identical to those used in the in vitro uptake assays. The suitability of DiD for biodistribution studies is supported by the fact that free dye in serum exhibits minimal fluorescence due to aggregate formation [49], a behavior we experimentally confirmed (Figure S4), ensuring that in vivo signal originates from the encapsulated dye. Additionally, DiD‐loaded NEs maintained stable fluorescence during serum incubation, confirming label integrity throughout the assay (Figure S4).

Whole‐body IVIS imaging showed a clear and selective fluorescence signal in the infected ears, confirming that GDQM‐NE‐DiD accumulates at the lesion site and colocalizes with the mCherry‐expressing parasites (Figure S5). Ex vivo imaging further validated this targeted accumulation and enabled quantitative comparison across organs, revealing strong uptake in the ears, despite their distal location, likely facilitated by local inflammation (Figure 9A,B). The liver, as the primary clearance organ, displayed the highest total fluorescence. However, when normalized to tissue area, the ears showed the most prominent signal, reflecting preferential accumulation at the infection site. The spleen exhibited only low fluorescence levels, consistent with the limited retention of small nanoparticles, which are below the size needed to be efficiently trapped in splenic filtration sites (<150 nm) [50]. Notably, a transient signal appeared in the kidneys at 24 h—but not at later time points—suggesting that a fraction of the NEs may undergo early renal elimination, likely facilitated by their small hydrodynamic diameter [51]. After 48 h, the fluorescence in the ears progressively declined, indicating partial clearance of the nanoparticles over time (Figure 9B).

To assess whether the nanoparticles were internalized by specific immune cell populations rather than remaining extracellular, we performed flow cytometry on cells from the ears and auricular dLNs at 24, 48, and 96 h post‐injection. Gating strategies are detailed in the (Figures S6 and S7). In the ears, neutrophils, macrophages, and DCs efficiently internalized the NEs. The fraction of double‐positive cells (NE^+^/mCherry^+^) was substantial across all three populations, with macrophages showing the highest levels, followed by DCs and neutrophils, demonstrating efficient co‐localization with the parasite and successful intracellular delivery of GDQM (Figure 9C,D). However, despite this efficient lesion accumulation and uptake by lesion‐infiltrating immune cells, CD86^+^ cells were not clearly detected in the ears (Figure 9E). This dissociation between delivery and activation suggests that local factors within established lesions may limit cellular responsiveness to TLR7 stimulation.

In auricular dLNs, where infection is minimal, B cells, myeloid cells, and pDCs efficiently internalized NEs, reaching maximal levels at 24 h and gradually declining thereafter (Figure 9F). In contrast to the lack of detectable CD86 induction in infected ears, GDQM‐NEs increased CD86 expression in these dLN populations, with levels peaking at 24 h (Figure 9G).

### 3.8 Nanoencapsulation Limits Early Systemic Inflammation Induced by GDQM

**FIGURE 10:**
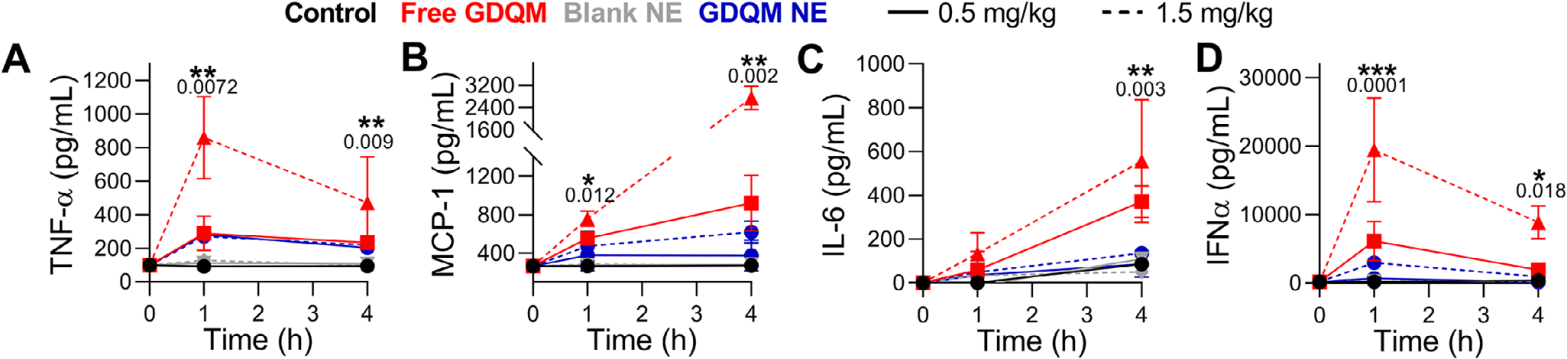
Plasma cytokine kinetics following intravenous administration of free GDQM, GDQM‐NE and blank NE. Plasma levels of TNFα (A), MCP‐1 (B), IL‐6 (C), and IFNα (D) were measured at 1 and 4 h post‐injection in mice treated with free GDQM (0.5 and 1.5 mg/kg), GDQM‐loaded nanoemulsions at matched doses, blank nanoemulsions, or left untreated. Data are shown as mean ± SD (*n* = 3–6). Solid lines indicate the 0.5 mg/kg dose, whereas dashed lines represent the 1.5 mg/kg dose. *p*‐values were calculated using the Kruskal–Wallis test followed by Dunn's multiple comparisons test relative to untreated controls at each time point; H = 16.85 for panel A, H = 14.83 for panel B, H = 15.93 for panel C, and H = 16.02 for panel D. Asterisks above columns indicate statistically significant differences relative to untreated controls: ^*^ *p* < 0.05, ^**^ *p* < 0.01, ^***^ *p* < 0.001, ^****^ *p* < 0.0001.

Uncontrolled systemic exposure to TLR7 agonists rapidly triggers cytokine release, limiting their clinical applicability. To assess whether nanoencapsulation modulates this response, early plasma cytokines were quantified following intravenous GDQM administration. Mice were assigned to seven groups, including untreated controls, free GDQM at two doses (0.5 and 1.5 mg/kg), GDQM‐loaded NEs at matched doses, and corresponding blank NEs administered at equivalent lipid concentrations to control for vehicle‐related effects (50 and 150 mg/kg of lipids). Cytokines were measured at 1 and 4 h post‐injection. Plasma levels of TNFα, MCP‐1, IL‐6, and IFNα were quantified (Figure 10A–D), as key mediators of systemic inflammatory responses downstream of TLR7 activation.

Free GDQM at 1.5 mg/kg induced a marked systemic cytokine surge, with rapid peaks in TNFα and IFNα and sustained increases in MCP‐1 and IL‐6. In contrast, encapsulated GDQM elicited only minimal, non‐significant responses despite equivalent dosing, while blank nanoemulsions were inert. Thus, nanoencapsulation effectively uncouples systemic exposure from acute inflammatory toxicity, preventing the cytokine surge associated with free GDQM.

### 3.9 GDQM Treatment Drives Parasite Control Accompanied by Concurrent Effector and Regulatory Immune Responses

**FIGURE 11:**
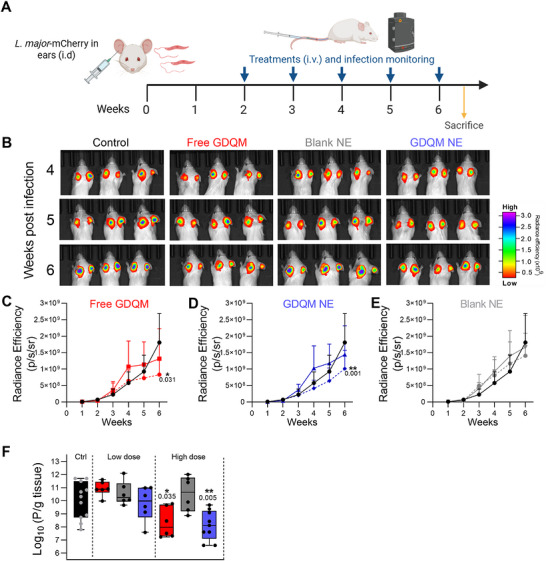
Therapeutic effect of free GDQM, GDQM‐loaded NEs, and blank NEs in a murine cutaneous leishmaniasis ear model. BALB/c mice were intradermally infected in the ear with 10^4^ metacyclic promastigotes of *Leishmania major*‐mCherry. (A) Experimental timeline: beginning two weeks post‐infection, mice received weekly intravenous administrations of free GDQM (0.5 and 1.5 mg/kg, low and high dose, respectively), GDQM‐loaded NEs at matched doses, or blank NEs at equivalent lipid concentrations; untreated mice were included as controls. (B) Representative fluorescence images at selected timepoints post‐infection (high dose). (C–E) Quantification of lesion fluorescence intensity over time. Red lines correspond to free GDQM, blue lines to GDQM‐loaded NEs, and grey lines to blank NEs. Dashed lines indicate high dose, whereas solid lines indicate low dose. (F) Parasite burden in ears at endpoint, determined ex vivo by limiting dilution assay. Data represent mean ± SD (*n* = 6–12). *p*‐values were calculated using the Kruskal–Wallis test followed by Dunn's multiple comparisons test relative to untreated controls at the final time point; H = 3.13 for panel C and H = 6.28 for panel F. Asterisks above data points or columns indicate statistically significant differences relative to untreated controls: ^*^ *p* < 0.05, ^**^ *p* < 0.01, ^***^ *p* < 0.001, ^****^ *p* < 0.0001.

Given the robust pro‐inflammatory and M1‐like activity induced by GDQM in vitro, together with the efficient in vivo accumulation and cellular co‐localization of NEs within infected lesions, we next compared the therapeutic performance of free and encapsulated GDQM in the established CL model. Blank NEs were also included as controls, based on their previously observed immunomodulatory effects in vitro, to account for potential carrier‐driven contributions to the immune response. Mice were allocated to seven experimental conditions, including untreated controls, free GDQM at two doses (0.5 and 1.5 mg/kg, once weekly), GDQM‐loaded NEs at matched doses, and blank NEs at equivalent lipid concentrations (Figure 11A). A weekly administration schedule was selected based on commonly used therapeutic vaccination regimens in this model [52, 53] and to avoid TLR7 desensitization associated with closely spaced dosing, as responsiveness has been shown to recover only after ≥7 days [54].

Disease progression was longitudinally monitored by in vivo fluorescence imaging, which revealed a clear dose‐dependent effect. A significant delay in parasite expansion was observed exclusively in the high‐dose (1.5 mg/kg) groups, both for free GDQM and GDQM‐loaded NEs, whereas low‐dose treatments and blank NEs had no measurable impact (Figure 11B–E). Thus, as observed in vitro, the partial immunomodulatory activity of the blank carrier did not translate into meaningful parasite control. In contrast, reduction in lesion size was observed exclusively in the high‐dose free GDQM group (Figure S8). Consistently, endpoint limiting dilution assays revealed a ∼2‐log reduction in parasite burden in infected ears from both high‐dose free and nanoencapsulated GDQM‐treated mice, while blank NEs had no effect. Thus, nanoencapsulation maintained the antileishmanial activity of GDQM but did not confer superior in vivo efficacy over the free compound. Parasite loads in dLNs remained low and unchanged across all groups (*data not shown*) (Figure 11F).

**FIGURE 12:**
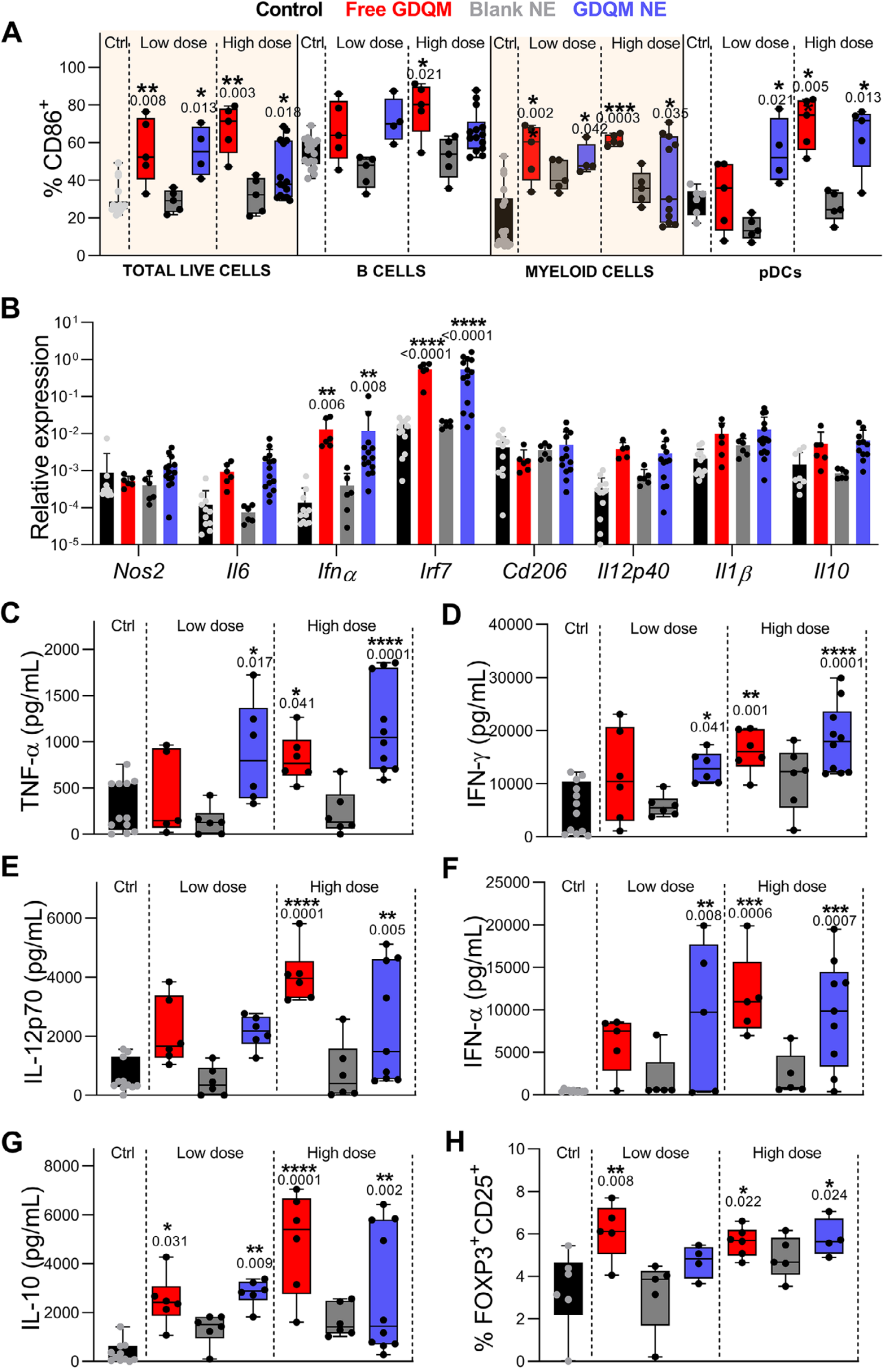
Immune response in auricular dLNs following treatment with free GDQM, GDQM‐loaded NEs, and blank NEs in *L. major*–infected mice. BALB/c mice were intradermally infected in the ear with 1 × 10^4^ metacyclic *L. major*‐mCherry promastigotes and treated once weekly for 5 weeks intravenous administrations of free GDQM at low (0.5 mg/kg) and high (1.5 mg/kg) doses, GDQM‐loaded NEs at matched low and high doses, or corresponding blank NEs at equivalent lipid concentrations. Untreated mice were included as controls. (A) Proportion of CD86^+^ antigen‐presenting cells. (B) Gene‐expression analysis of selected genes. (C–G) Cytokine concentrations in the supernatant of dLNs cells restimulated for 24 h with SLA: (C) IFN‐γ, (D) IL‐12p70, (E) TNF‐α, (F) IFN‐α, and (G) IL‐10. H) Frequency of FOXP3^+^‐CD25^+^ regulatory T cells in dLNs cells restimulated with SLA. Data represent mean ± SD (*n* = 6–12). *p*‐values were calculated using one‐way ANOVA followed by Tukey's multiple comparisons test relative to untreated controls; F = 11.02 for panel A, F = 8.6 for panel C, F = 5.97 for panel D, F = 9.10 for panel E, F = 7.01 for panel F, F = 7.24 for panel G, and F = 4.35 for panel H. Asterisks above columns indicate statistically significant differences relative to untreated controls: ^*^ *p* < 0.05, ^**^ *p* < 0.01, ^***^ *p* < 0.001, ^****^ *p* < 0.0001.

To further characterize the immune response elicited by the treatment, we analyzed the dLNs at endpoint (Figure 12). Both free and nanoencapsulated GDQM induced an increased proportion of CD86^+^ cells across the total viable population at both doses, with a similar trend observed in B cells, myeloid cells, and pDCs, indicating broad activation of antigen‐presenting populations (Figure 12A). Transcriptional profiling revealed that *Ifna* and *Irf7* were the only genes showing significant upregulation, and this was restricted to the high‐dose groups (Figure 12B), while no significant changes were detected at the low dose (*data not shown*), consistent with activation of canonical TLR7‐associated signaling pathways. Functionally, cytokine production following ex vivo restimulation with SLA was assessed in dLN cells. IFN‐γ, IL‐12p70 and TNF‐α were markedly elevated predominantly in the high‐dose groups, with comparable trends between free and nanoencapsulated GDQM (Figure 12C–E). In parallel, IFN‐α levels were also increased under these conditions (Figure 12F), consistent with the transcriptional signature observed. IL‐10 was likewise increased in both free and nanoencapsulated GDQM groups, with a more pronounced effect at the high dose (Figure 12G), accompanied by an increased frequency of FOXP3^+^CD25^+^ regulatory T cells (Figure 12H); the gating strategy is detailed in Figure S9. This mixed inflammatory/regulatory profile likely reflects a compensatory mechanism that restrains excessive inflammation. While it supports some degree of parasite control, it may also limit the overall therapeutic impact, consistent with the modest reduction in parasite burden and the absence of lesion improvement.

### 3.10 Encapsulation Mitigates Systemic and Neuroinflammatory Toxicity Effects Associated With Repeated GDQM Administration

**FIGURE 13:**
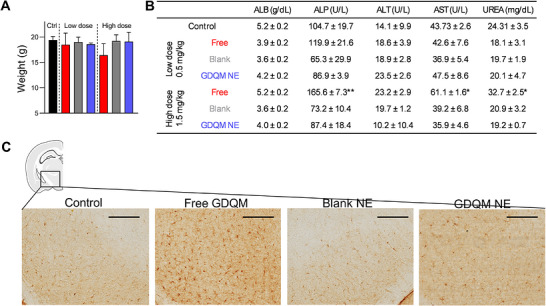
Systemic safety profile following repeated administration of free GDQM, GDQM‐loaded NEs, and blank NEs in *L. major*–infected mice. BALB/c mice were intradermally infected and treated with weekly intravenous administrations of free GDQM (0.5 and 1.5 mg/kg, low and high dose, respectively), GDQM‐loaded NEs at matched doses, or blank NEs at equivalent lipid concentrations. Systemic toxicity was assessed at the end of the treatment period. (A) Body weight at endpoint. (B) Serum biochemical parameters, including liver and renal function markers (AST, ALT, ALP, urea, albumin). Data in A and B are presented as mean ± SD, with *n* = 3 mice per condition. *p*‐values were calculated using the Kruskal–Wallis test followed by Dunn's multiple comparisons test relative to untreated controls. No significant differences were observed in endpoint body weight (A; H = 0.41). For serum biochemical parameters (B), Kruskal–Wallis test statistics were as follows: AST, H = 2.65; ALT, H = 0.32; ALP, H = 3.21; urea, H = 2.43; and albumin, H = 0.25. In B, Asterisks above columns indicate statistically significant differences relative to untreated controls: ^*^ *p* < 0.05, ^**^ *p* < 0.01, ^***^ *p* < 0.001, ^****^ *p* < 0.0001. (C) Representative 20x images of Iba1 immunohistochemistry in brain sections of the *substantia nigra*, illustrating microglial activation. Scale bar: 200 µm.

The systemic administration of TLR7 agonists has been associated with hepatotoxicity and, in some cases, signs of neurotoxicity [55]. Accordingly, following repeated dosing in the efficacy study, systemic safety was evaluated at the end of the treatment period (Figure 13). Body weight remained largely stable across groups, with a slight reduction observed in mice treated with high‐dose free GDQM (1.5 mg/kg), although this did not reach statistical significance (Figure 13A).

Serum biochemical analysis showed that most parameters remained within normal physiological ranges. However, mice treated with high‐dose free GDQM exhibited significant increases in AST, ALP, and urea compared to untreated controls (Figure 13B), whereas no significant alterations were observed in mice receiving GDQM‐loaded nanoemulsions at either dose. Notably, ALT levels remained unchanged, indicating the absence of overt hepatocellular injury, while the elevation of AST and ALP is consistent with systemic inflammatory stress and mild hepatic involvement [56]. Additional markers, including GGT, CRP, bilirubin, and creatinine, were unchanged across groups (*data not shown*).

Moreover, mice receiving high‐dose free GDQM displayed behavioral alterations during the final week of treatment, including circling movements and impaired coordination. To further investigate this phenotype, brain sections from the *substantia nigra*, a region involved in motor control, were analyzed by Iba1 immunohistochemistry. An increased number of Iba1^+^ cells was observed in the high‐dose free GDQM group, whereas mice treated with GDQM‐loaded NEs showed markedly fewer Iba1^+^ cells, suggesting a neuroinflammatory component associated with the observed behavioral alterations (Figure 13C).

Although these analyses were performed with a limited number of animals per group (*n* = 3, as indicated in the figure caption), the consistency of the biochemical, behavioral, and histological findings supports the protective effect of nanoencapsulation against GDQM‐associated systemic and neuroinflammatory toxicity.

## 4 Discussion

The present study describes a NE formulation loaded with the TLR7 agonist GDQM that effectively uncouples immunostimulatory activity from systemic toxicity. Similar nanomaterial‐based strategies have recently been explored for macrophage‐targeted TLR7/8 immunomodulation [57, 58]. Compared with the free agonist administered at the same dose (1.5 mg/kg), GDQM‐NE markedly reduced circulating pro‐inflammatory cytokines and prevented microgliosis, highlighting its improved safety profile (Figures 10 and 13). Following intravenous administration, the NE exhibited a high degree of colocalization with parasites in dermal skin lesions, together with enhanced uptake and increased CD86 expression in antigen‐presenting cells within the auricular dLNs (Figure 9). Ex vivo re‐stimulation with SLA antigen elicited a Th1‐biased immune response, characterized by elevated production of IFN‐γ and IL‐12p70. At the same time, regulatory features were evident, including increased IL‐10 production and expansion of FoxP3^+^ regulatory T cells (Figure 12). This mixed immune profile was associated with an approximately 2‐log reduction in parasite burden (Figure 11F). However, this reduction was comparable to that achieved with high‐dose free GDQM and did not translate into superior lesion‐size control. Therefore, the main therapeutic advantage of GDQM‐NE is the markedly safety profile together with the preservation of antileishmanial activity.

Despite efficient parasite‐NE colocalization within lesions, no increase in CD86 expression was detected at the site of infection, in contrast to the activation observed in dLNs (Figure 9). This suggests that macrophages within established lesions fail to fully respond to TLR7 stimulation in situ. This observation is particularly relevant because *Leishmania* is an intracellular parasite that resides and persists within macrophages, which in chronic lesions are typically skewed toward an M2‐like, immunosuppressive phenotype [4]. In vitro, the GDQM‐loaded NE induced a robust M1‐like phenotype, as evidenced by changes in gene expression, cytokine secretion, and metabolomic profiling, and promoted dose‐dependent parasite killing (Figures 3, 4, 5, 6, 7, 8). This polarization and leishmanicidal activity were strongly shaped by the cytokine milieu but remained partially preserved even under IL‐4 exposure, suggesting that GDQM‐NE can overcome, at least in part, M2‐skewing signals. The discrepancy between in vitro and in vivo responses underscores the influence of the complex dermal microenvironment in chronic CL. Within established lesions, multiple non‐exclusive factors constrain macrophage activation, including local immunosuppressive mediators, pH alterations, and metabolic tissue‐specific conditions [59] may limit the ability of macrophages to fully respond to TLR7 stimulation despite efficient delivery and parasite targeting by the NE.

Building on this, IL‐10 production was consistently observed in vitro (Figure 5C), indicating the activation of intrinsic negative feedback mechanisms downstream of TLR7 signaling, likely serving to avoid excessive inflammation while potentially could constraining maximal parasite clearance. This regulatory component was further reinforced in vivo, where GDQM treatment induced an expansion of regulatory T cells (FoxP3^+^), particularly at the lower dose (0.5 mg/kg), whereas the higher dose (1.5 mg/kg) favored a stronger pro‐inflammatory cytokine profile (IFN‐γ, IL‐12, TNF‐α) (Figure 12). Crucially, significant parasite reduction was only observed in vivo at the higher doses of 1.5 mg/kg (Figure 11), highlighting that effective clearance requires conditions in which Th1‐proinflamatory bias overcome regulatory constraints [60, 61]. This dual inflammatory–regulatory behavior has been widely reported for TLR7/8 agonists (and other TLR agonists), where immune activation is intrinsically counterbalanced by feedback mechanisms that may compromise the therapeutic efficacy of these immunotherapies [40, 62, 63]

Thus, although NEs reduce systemic toxicity and permit higher dosing, the benefit in efficacy of this TLR‐based immunotherapy seems to be constrained by directly TLR macrophage intrinsic regulatory mechanisms and T cell–mediated feedback. The dominant influence of cytokine (IFN‐γ vs. IL‐4) observed in the antileishmanial activity observed in vitro for GDQM‐NEs (Figure 8), together the classical three‐signal model of macrophage activation and the dose‐dependent IL‐10 production and expansion of FoxP3 regulatory T cells, pointed out that the balance between pro‐inflammatory and regulatory responses ultimately determines therapeutic outcome. Accordingly, strategies to improve therapeutic performance should focus on modulating this balance rather than simply increasing the dose of GDQM‐NE. Combining TLR agonists with immune checkpoint inhibitors may help overcome suppressive pathways and promote more effective parasite elimination, although this could impair tissue repair [4]. Alternatively, combining TLR7 activation with conventional drugs may reduce lesion burden and modulate the microenvironment without compromising skin regeneration. Additionally, pairing TLR7 stimulation with relevant antigens may further focus and amplify antigen‐specific responses [52, 64, 65]. Altogether, this combinatorial approach addresses both the insufficient immune priming and the regulatory mechanisms observed in our model, leading to a more effective therapeutic response.

Beyond the contribution of the agonist, the role of the nanocarrier itself should also be considered. In vitro, blank NEs induced a partial M1‐like immunometabolic profile, reflecting the intrinsic immunomodulatory properties of the lipid components used in this formulation, including phosphatidylcholine, stearate‐based surfactants, and triglycerides [66, 67]. However, this activation did not translate into antimicrobial effector functions or parasite killing, either in vitro or in vivo (Figures 8 and 11 and 12), suggesting that carrier‐driven activation alone is insufficient to promote effective microbicidal responses, particularly within complex tissue environments and at the tested dose in mice (50 or 150 mg/kg). At the same time, these findings highlight the potential of the nanocarrier as an active modulator of the immune response and support further optimization of the delivery system. For instance, incorporating lipids with stronger or more specific immunomodulatory properties (e.g., phosphatidylserine‐ or sphingolipid‐derived components) could help fine‐tune the balance between activation and regulation [68], ultimately improving therapeutic efficacy. In this context, it is also important to reconsider the criteria for optimal drug loading, which may not necessarily correspond to maximal payload, but rather to a formulation that preserves or enhances the immunomodulatory contribution of the carrier itself.

During the course of this work, we also identified key variables that may influence therapeutic efficacy, including dose, schedule, and route of administration. Dose selection was guided by previously reported regimens for TLR7/8 agonists, such as R848 (1.5–3 mg/kg), as no reliable in vitro–to–in vivo dose translation has been established for this class of compounds. The weekly administration schedule was chosen based on commonly used therapeutic vaccination protocols in this disease model [52, 65] and to mitigate the risk of TLR tolerance and/or T cell exhaustion associated with frequent or intense stimulation, as longer dosing intervals (≥7 days) have been shown to partially restore cytokine responsiveness [54].

Regarding the route of administration, intravenous delivery enabled substantial accumulation of GDQM‐NE in dermal lesions (Figure 9). Alternative routes, such as intralesional administration, could place the formulation in closer proximity to the parasite niche, while encapsulation may help limit the rapid diffusion and systemic toxicity of free agonists. Nonetheless, this approach may not be suitable for certain clinical manifestations, such as diffuse cutaneous leishmaniasis (DCL) or post–kala‐azar dermal leishmaniasis (PKDL), where the presence of multiple, widely distributed lesions containing heavily infected M2 macrophages reduces the feasibility of localized treatment [69].

Another important consideration for TLR‐based therapies is the difference between murine and human immune responses. TLR7 expression differs significantly between species, with important implications for translation. In mice, TLR7 is broadly expressed across several immune cell populations, including pDCs, B cells, conventional dendritic cells, and notably monocytes/macrophages, enabling direct activation of innate effector functions. In contrast, in humans, TLR7 expression is more restricted, being predominantly found in pDCs and, to a lesser extent, B cells, with limited and variable expression in monocytes and macrophages [70]. As a result, TLR7‐mediated responses in humans are likely to rely more on pDC‐derived type I interferons, whereas in mice, both direct macrophage activation and broader innate immune engagement contribute to the overall response. These interspecies differences should be carefully considered when interpreting preclinical results and translating TLR7‐targeted therapies to the clinical setting. Notably, recent studies in experimental models of leishmaniasis have suggested that type I interferons may impair parasite control by promoting regulatory pathways and dampening protective Th1 responses [71, 72, 73]. In humans, type I interferon signatures have been detected in lesional tissues of CL patients; however, these responses coexist with strong inflammation and do not correlate with effective parasite control [74].

## 5 Conclusions

We provide a comprehensive assessment of the holistic pro‐inflammatory (M1) effects of GDQM‐loaded NEs on macrophages, examining everything from transcriptional changes to effector functions and metabolic reprogramming. GDQM‐NEs efficiently reach infected macrophages in the deep layers of the skin and co‐localize with *Leishmania*, addressing one of the major challenges in CL: targeted delivery to the infection niche. Importantly, nanoencapsulation enables this targeted accumulation while markedly reducing systemic exposure and limiting inflammation‐associated toxicity compared with the free compound, thereby improving the overall safety profile of the immunostimulant. Despite this improved safety and effective delivery, in vivo therapeutic efficacy remained partial, with a reduction in parasite burden of 2‐log but no clear improvement in lesion outcome, underscoring that correct biodistribution and macrophage targeting alone are insufficient to overcome the complex immunological constraints of established lesions. Ultimately, the local immune landscape dictates therapeutic outcome, and factors such as timing of intervention, route of administration, and rational combination strategies are likely required to fully exploit the therapeutic potential of TLR‐based immunomodulation. Together, these findings highlight the GDQM‐NE platform as a versatile and safer delivery system for immunomodulatory agents, with broad applicability beyond cutaneous leishmaniasis, particularly in contexts where targeted immune activation must be balanced against systemic toxicity.

## Author Contributions

**Carmen Palomino‐Cano**: writing – review & editing, writing – original draft, visualization, validation, methodology, investigation, formal analysis, data curation. **M. Carmen Mera‐Delgado**: investigation. **Esther Moreno**: investigation, writing – review, and editing. **Esther Larrea**: investigation and resources. **Lecnia Aguirre**: investigation. **Jonathan M. Zannatta**: investigation. **Sónia Barros‐Carvalho**: investigation. **Iola F. Duarte**: investigation, analysis, writing – review, and editing. **Ricardo Silvestre**: writing – review, and editing, resources. **Javier Carrión**: supervision and resources. **Juan M. Irache**: supervision and resources. **Socorro Espuelas**: conceptualization, methodology, resources, supervision, funding acquisition, writing – review, and editing. All authors have approved the final version of the manuscript.

## Funding

This work was supported by the Spanish Ministry of Science, Innovation and Universities (PID2023‐148406OB‐I00). C.P.‐C. gratefully acknowledges the Instituto de Salud Tropical of the University of Navarra, the University of Navarra, and the Erasmus+ program for supporting her predoctoral fellowship and the stay in Braga. Metabolomic studies were developed within the scope of the project UID/50006/2025, Laboratório Associado para a Química Verde ‐ Tecnologias e Processos Limpos (LAQV‐REQUIMTE; DOI 10.54499/UID/50006/2025), and projects UID/06304/2025 (https://doi.org/10.54499/UID/06304/2025) and LA/P/0050/2020 (https://doi.org/10.54499/LA/P/0050/2020) financed by national funds through FCT – Fundação para a Ciência e a Tecnologia. Additional funding was provided by the European Regional Development Fund (FEDER) through the COMPETE 2030 Programme (project COMPETE2030‐FEDER‐00735300). The NMR spectrometer is part of the National NMR Network (PTNMR), partially supported by Infrastructure Project No. 022161 (cofinanced by FEDER through COMPETE 2020, POCI, and PORL and FCT through PIDDAC).

## Ethics Statement

All experimental procedures were approved by the Animal Experimentation Ethics Committee of the University of Navarra (protocol number 123‐21) and carried out in accordance with current European regulations on the use of animals in research (Directive 2010/63/EU).

## Conflicts of Interest

The authors declare no conflicts of interest.

## Supporting information




**Supporting File**: adhm71415‐sup‐0001‐SuppMat.docx.

## Data Availability

The data that support the findings of this study are available from the corresponding author upon request.
