# Supplementary material for: Gardiquimod Nanoemulsion Targets Cutaneous Leishmaniasis Lesions Reducing Systemic Toxicity and Parasite Burden
Source: Adv Healthc Mater. 2026 Jul 6;15(30):e71415. doi: 10.1002/adhm.71415 (PMC13474118; doi:10.1002/adhm.71415)
Supplement: Supplementary file 1 [file ADHM-15-0-s001.docx]

**SUPLEMENTARY MATERIAL**

**Table S1**.- Sequences of the primers used for RT-qPCR experiments

| Gene | Sense primer (5´-3´) | Antisense primer (5´-3´) |
| --- | --- | --- |
| *Actin* | CGCGTCCACCCGCGAG | CCTGGTGCCTAGGGCG |
| *Leishmania 18 S* | CCAAAGTGTGGAGATCGAAG | GGCCGGTAAAGGCCGAATAG |
| *Arg1* | TGGGGAAAGCCAATGAAGAG | AGGAGAAAGGACACAGGTTG |
| *Inos* | TCCTACACCACACCAAACTG | AATCTCTGCCTATCCGTCTC |
| *Cd206* | GATTGTGGAGCAGATGGAA | TTTGTCGTAGTCAGTGGTGG |
| *Tnfα* | CTTCCAGAACTCCAGGCGGT | GGTTTGCTACGACGTGGG |
| *Il1β* | GCCACCTTTTGACAGTGATG | TAATGGGAACGTCACACACC |
| *Il6* | ACAAAGCCAGAGTCCTTCAG | TGGATGGTCTTGGTCCTTAG |
| *Il10* | GGACAACATACTGCTAACCG | AATCACTCTTCACCTGCTCC |
| *Il12p40* | AGATGAAGGAGACAGAGGAG | GGAAAAAGCCAACCAAGCAG |
| *Irf7* | AAGCATTTCGGTCGTAGGG | CTTCCAGCCTCTTCGCTCT |
| *Ifnα* | AAAGAAATGTAAGAAAGCTTT | TACACTTTGGCTCAGGACTCA |
| *Hk1* | GAAAGGAGACCAACAGCAGA | TTCGTTCCTCCGAGATCCAAG |

**Figure S1.- Representative scanning electron microscopy image of GDQM-NEs**. Scale bar: 200 nm.

**Figure S2.-** **Uptake of GDQM-loaded NEs by BMDMs *in vitro***. Representative dot plot (SSC vs DiD fluorescence) showing increased cell complexity and DiD signal upon nanoparticle uptake.

**Figure S3.- Seahorse metabolic profiling of BMDMs treated with GDQM-NE.** Macrophages were treated with GDQM-NE (10 µM GDQM) for 48 h in the presence or absence of costimuli (IL-4 or IFN-γ). (A) Oxygen consumption rate (OCR) over time during the Seahorse XF Mito Stress Test, indicating the sequential injection of oligomycin, FCCP, rotenone/antimycin A, and 2-DG. (B) Basal respiration. (C) Non-mitochondrial respiration. (D) Proton leak. (E) Non-glycolytic acidification. Statistical significance relative to untreated controls was determined by Kruskal–Wallis test followed by Dunn’s multiple comparisons test (*p < 0.05, **p < 0.01, ***p < 0.001, ****p < 0.0001).

**Figure S4.- Suitability of DiD labeling for biodistribution studies and serum stability of GDQM-NEs**. **A)** Fluorescence images and quantitative analysis confirming the suitability of DiD for tracking GDQM-NEs. Representative IVIS Spectrum images show DiD in DMSO or spiked into mouse serum at different concentrations (left), demonstrating strong signal in DMSO and negligible fluorescence when free dye aggregates in serum. Quantification of radiance efficiency for each condition is shown on the right. B) Assessment of nanoparticle stability in serum. Fluorescence images of GDQM-NEs (1% DiD) redispersed in fresh mouse serum and incubated for the indicated time points (left). Corresponding fluorescence intensity measurements (right) show stable signal over time, indicating preserved integrity of the DiD-loaded NEs during serum incubation. All fluorescence measurements were acquired using the IVIS Spectrum imaging system, and data are presented as mean ± SD of two independent experiments.

**Figure S5.- Whole-body biodistribution of GDQM-NE-DiD 24 h after intravenous administration**. Representative IVIS Spectrum whole-body fluorescence images of BALB/c mice acquired 24 h after injection of GDQM-NE-DiD (1 mg/kg, i.v.). Mouse #1 corresponds to an untreated, uninfected control. Mouse #2 represents an infected control (*L. major*-mCherry, ear lesion) that did not receive nanoparticles. Mice #3–5 were infected and administered GDQM-NE-DiD. Fluorescence signals depict the in vivo biodistribution of DiD-labeled NEs, highlighting their accumulation in infected dermal sites. Images were acquired using the IVIS Spectrum imaging system under identical exposure settings.

**Figure S6.- Gating strategy for myeloid cells and NE-DiD/*L. major*-mCherry colocalization in ear tissue**. Flow cytometry gating used to identify neutrophils, dendritic cells, and macrophages from ear samples and to assess intracellular colocalization of NE-DiD with *L. major*-mCherry. All samples were acquired under identical cytometer settings.

**Figure S7.- Gating strategy for auricular draining lymph nodes**. Flow cytometry gating was used to identify B cells (B220⁺, CD11c⁻), plasmacytoid dendritic cells (pDCs, B220⁺, CD11c⁺), and myeloid antigen-presenting cells (B220⁻, MHC II⁺). Within each gated subset, the percentage of NE-DiD⁺ cells and CD86⁺ activated cells was subsequently quantified (not shown). All samples were processed and acquired under identical conditions.

**Figure S8.- Evolution of lesion size in a murine cutaneous leishmaniasis ear model following treatment with free GDQM, GDQM-loaded NEs, and blank NEs.** BALB/c mice were intradermally infected in the ear with 10⁴ metacyclic promastigotes of *Leishmania major*-mCherry and treated as described in Figure 11. Lesion size was measured over time using calipers. Red lines correspond to free GDQM, blue lines to GDQM-loaded NEs, and grey lines to blank NEs. Dashed lines indicate high dose (1.5 mg/kg), whereas solid lines indicate low dose (0.5 mg/kg). Data represent mean ± SD (n = 6–12). Statistical significance relative to untreated controls at the final time point was determined using the Kruskal–Wallis test followed by Dunn’s multiple comparisons test (H = 2.54). Asterisks indicate statistical significance relative to untreated controls

**Figure S9.- Gating strategy for Treg analysis in auricular draining lymph nodes**. Flow cytometry gating used to identify CD4⁺ T cells and the regulatory T-cell subset (CD4⁺ FOXP3⁺) in auricular draining lymph nodes. All samples were processed and acquired under identical conditions.
